# Implementing front-of-pack nutrition warning labels in Mexico: important lessons for low- and middle-income countries

**DOI:** 10.1017/S1368980023001441

**Published:** 2023-10

**Authors:** Eric Crosbie, Margarita Gabriela Otero Alvarez, Michelle Cao, Lesly Samara Vejar Renteria, Estefania Rodriguez, Ana Larrañaga Flota, Angela Carriedo

**Affiliations:** 1 School of Public Health, University of Nevada Reno, Reno, NV 89557, USA; 2 Ozmen Institute for Global Studies, University of Nevada Reno, Reno, NV, USA; 3 Instituto Nacional de Salud Pública, Mexico City, Mexico; 4 University of Sussex, Brighton, UK; 5 Department of Health, University of Bath, Bath, UK; 6 World Public Health Nutrition Association, London, UK

**Keywords:** Nutrition, Labelling, Mexico, Policy implementation

## Abstract

**Objective:**

To analyse the implementation of front-of-pack nutrition labelling (FOPNL) in Mexico.

**Design:**

Review of publicly accessible documents, including legislative websites, news sources, and government, intergovernmental, and advocacy reports. Usage of the policy cycle model to analyse the implementation and evaluation stages of Mexico’s General Health Law, amended with FOPNL (2019–2022).

**Results:**

In October 2019, the government published a draft modification of the Norma Oficial Mexicana (Official Mexican Standard) to regulate and enforce a new FOPNL warning label system. A 60-d public consultation period followed (October–December 2019), and the regulation was published in March 2020 and implementation began in October 2020. An analysis of nine key provisions of the Standard revealed that the food and beverage industry and its allies weakened some original provisions including health claims, warnings for added sweeteners and display areas. On the other hand, local and international public health groups maintained key regulations including the ban on cartoon character advertisements, standardised portions and nutrient criteria following international best practices. Early implementation appears to have high compliance and helped contribute to reformulating unhealthy products. Continued barriers to implementation include industry efforts to create double fronts and market their cartoon characters on social media and through digitalised marketing.

**Conclusion:**

Early success in implementing the new FOPNL system in Mexico was the result of an inclusive and participatory regulatory process dedicated to maintaining public health advances, local and international health advocacy support, and continued monitoring. Other countries proposing and enacting FOPNL should learn from the Mexican experience to maintain scientifically proven best practices, counter industry barriers and minimise delays in implementation.

An unhealthy diet is one of the leading causes of non-communicable diseases (NCD), including type 2 diabetes, CVD and cancer, contributing to death and disability worldwide^(1)^. The overconsumption of ultra-processed foods and drinks has played an important role in the increase of overweight/obesity and NCD and nutrition-related diseases globally^(1)^. To address these issues, the WHO recommends the implementation of effective and clear front-of-pack nutrition labelling (FOPNL) systems, which present nutrition information on the front of packaged foods and beverages that helps populations understand nutritional content to make healthier choices, reduce consumption of ultra-processed foods and drinks, and may generate a reformulation of food portfolios^(2)^.

Over the past decade, FOPNL policies have begun to spread rapidly worldwide, especially in Latin America^(3)^. This began in Chile (2012) followed by Peru (2013), Mexico (2014), Ecuador (2014), Bolivia (2017), Uruguay (2019), a re-designed FOPNL in Mexico (2020), Brazil (2020), Colombia (2021) and Argentina (2021)^(3)^. Several other countries in the region are considering the adoption of a mandatory FOPNL to help address the NCD epidemic in the region.

Many of these mandatory FOPNL policies have either been challenged or delayed by the food industry through domestic and international courts^(4–6)^. Learning from these experiences, governments are more aware and willing to protect policy spaces and follow key recommendations from intergovernmental organisations such as the WHO and health advocates^(3)^. Tracking this process not only reveals the pace of policy adoption and implementation^(3)^ but underscores how corporate political activity can influence the political process and undermine public health policies aimed at reducing the NCD epidemic.

Nowhere is the situation of NCD and nutrition-related diseases more paramount than in Mexico where the prevalence of obesity increased by nearly 50 % from 2000 to 2018 and rates for severe obesity nearly doubled^(7)^. Of particular concern is the impact of childhood overweight/obesity, which affects almost four out of ten children in the country^(7)^. In response, the Mexican government has implemented several policies to address this including increasing taxes on soda and restricting food marketing^(8)^. In October 2019, the Mexican government passed amendments to the General Health Law which established a FOPNL warning system which places octagon warning labels on products with high content of sugar, saturated fats, trans fats, calories, and salt and two warning captions on caffeine and non-caloric sweeteners on the front of food and drink packages (Fig. 1).

**Fig. 1:**
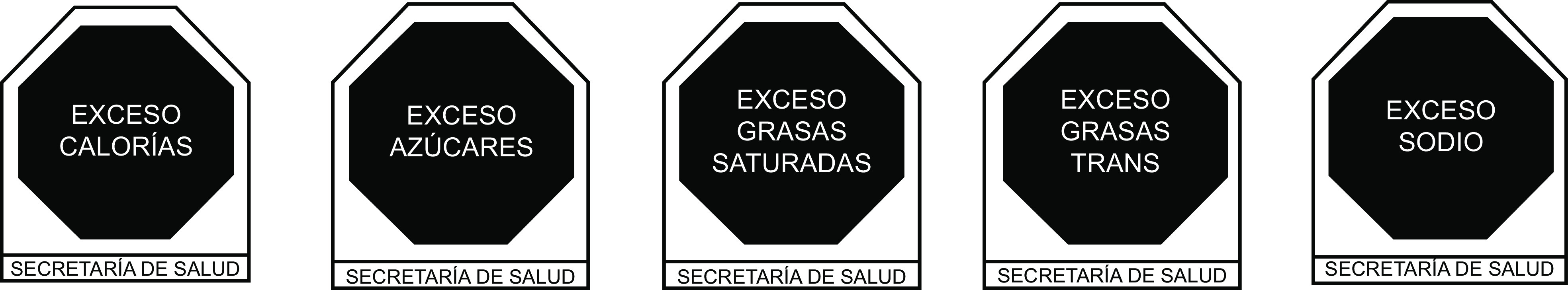
Example of front-of-pack nutrition label warnings in Mexico

While the legislative process to enact public health policies has been thoroughly studied^(9,10)^, including the passage of FOPNL in Mexico^(11)^, the implementation stage, involving implementing, enforcing and evaluating the law, has been overlooked and not studied with the same importance, especially in low- and middle-income countries^(12,13)^. This study aims to fill this gap by examining the early stages of implementation of FOPNL in Mexico and the emerging evaluations and outcomes. In particular, it examines (a) the regulation (guidelines for implementing the law), (b) monitoring, enforcement and compliance, (c) barriers and challenges to implementation, and (d) evaluating the policy. In doing so, this study looks to provide important lessons about implementation strategies and challenges to implementation for other low- and middle-income countries that are currently proposing and implementing FOPNL.

## Methods

### Data collection

Between November 2021 and May 2022, we reviewed publicly accessible documents available on Google, including media reporting, government and intergovernmental reports, public submissions by key stakeholders, and legislative and executive websites in Mexico. Standard snowball search methods were conducted in both English and Spanish beginning with key search terms, including ‘Mexico’, ‘labeling’, ‘warning label’, ‘beverage industry’, and specific supporting and opposing stakeholder groups. A total of thirty-four relevant documents were located and used for this analysis.

### Data analysis

To identify the key similarities and changes that occurred from approving to implementing FOPNL in Mexico, we first compared the initial draft modification in October 2020^(14)^ (before public consultation) to the Norma Oficial Mexicana (Official Mexican Standard, hereinafter referred to as Standard), which represent the mandatory technical changes required to implement and enforce the law, with the final Standard (after the consultation) that was published in March 2021^(15)^. Then we reviewed the executive branch summary report of public comments on the draft modification to NOM-051 (the Standard). A total of 117 provisions were reported on, of which we analysed nine key provisions (guidelines, directives, and rules ‘within’ laws and policies) on FOPNL based on a minimum requirement of five pages of discussion summary. Provisions that were not directly related to FOPNL or did not contain significant discussion were excluded. From these nine provisions, we identified and reviewed executive branch report summaries and actual submissions of eight supporting (public health advocates and allies) and eight opposing stakeholder groups (food and beverage industry and allies) (Table 1).

**Table 1:** Development and outcome of the Official Mexican Standards for front-of-pack nutrition labelling in Mexico (October 2019–March 2020)

Provisions	NOM draft (October 2019)^(14)^	Opposing arguments (October–December 2019)	Supporting arguments (October–December 2019)	Published NOM (March 2020)^(15)^	Outcome
Ban on health claims	Prohibited all claims and endorsements on products with FOPNL warnings from professional health associations	– Violates Federal Law for Consumer Protection Article 32 (establishes minimum requirement for the use of product endorsements)^(16,17)^ – Disqualifies credibility of a government entity (e.g. Health Ministry)^(18)^	– All information under Federal Law for Consumer Protection must be free of confusing or deceiving elements and state has legitimate public health intertest^(19–21)^ – Some medical association endorsements have conflicts of interest (e.g. Mexican Diabetes Federation receiving funding from Nestlé)^(20)^	Allowed any claims and endorsements on FOPNL from professional health associations only on products with no FOPNL warning labels	– Unchanged – MoE explanation: allowed in conformity with Federal Law for Consumer Protection to promote informed consumption – (Public health win)
Warning labels for added sweeteners	Required warning labels (black octagons) on products containing added sweeteners. The label would read ‘Contains Sweeteners. Avoid in Children’	– FDA and CODEX deem sweeteners as safe^(18,22,23)^ – Poses unnecessary obstacles to trade and violates TBT Agreement^(24)^ – Wording in warning label restricts consumption; exceeds authority of regulatory entity^(24)^ – Hinders reformulation^(23)^	– There is evidence indicating the detrimental impact of sweeteners^(19,20)^ – Visibility of warning labels is crucial, especially since these products are often marketed to children	Required warning captions (smaller text boxes) on products containing added sweeteners. The wording in the label changed to ‘Contains Sweeteners. Not Recommended for Children’	– Changed – MoE explanation: allowed in conformity with General Health Law Article 212 and based on evidence that sweeteners can instil a preference for sweet flavours among children – (Industry win)
Warning label display areas (simplified warning labels)	Allowed products with a display area ≤ 20 cm^2^ to include only one simplified warning label (a black octagon with the digit corresponding to the number of critical nutrients they contain)	– FOPNL warning labels interfere with the visibility of other elements on the product label^(24)^ – Products with a main display area < 20 cm are exempt from including nutrition facts and should therefore be exempt from the FOPNL warning labels^(24)^	– Imperative to establish narrower display area parameters for the simplified warning labels (i.e. decrease cutoff point to ≤ 10 cm^2^). Industry in Peru downsized products to evade FOPNL requirements^(22)^	Doubled the parameters and allowed products with a display area ≤ 40 cm^2^ to include only one simplified warning label (a black octagon with the digit corresponding to the number of critical nutrients they contain).	– Changed – MoE explanation: the dimensions proposed by the draft NOM would obstruct the visibility of the FOPNL warning labels – (Industry win)
Warning labels on multipacks	Required all individual products sold in multipacks to include the corresponding FOPNL warning labels (in addition to the outer package)	– Products in multipacks are required to include the label ‘Not for individual sale’ and thus should be exempt from FOPNL requirements^(22)^	N/A	Required only the outer package in multipacks to include the corresponding FOPNL warning labels	– Changed – MoE explanation: None – (Industry win)
‘Product does not contain FOPNL warning labels’ claims	Prohibited products from indicating that they are FOPNL-free	– Violates Federal Law for Consumer Protection Article 1 (requires clear and accurate information about the product’s content, composition and characteristics) ^(22)^	– Products can use these claims as advertising, to make their products appear healthier than they actually are. Industry in Peru uses this strategy^(24)^	Allowed products without FOPNL to include the text ‘No contiene sellos ni leyendas’ (the product does not contain warning labels) on their main packaging	– Changed – MoE explanation: None – (Industry win)
Placement of warning labels on products with a main display area ≤ 60 cm^2^	Required products – regardless of size – to include the appropriate FOPNL warning labels (octagons) strictly on the upper right corner of their main display area	– There is a lack of evidence to support the use of FOPNL warnings^(24)^ – Placement of warning labels is irrelevant^(22,24)^ – Producers should be able to place FOPNL warning labels anywhere on the product’s main display area^(17)^	– Based on Chile’s experience, the regulation should establish a protected zone around the warning labels to prevent producers from including additional messages^(24)^	Allowed products with a main display area ≤ 60 cm^2^ to include the corresponding FOPNL elements anywhere on the label’s main display area	– Changed – MoE explanation: the placement of the warning labels on the main display area is intended to promote informed decision-making – (Industry win)
Nutrient profile model	Used the Pan American Health Organization (PAHO) Nutrient Profile Model as the guiding criteria for the implementation of FOPNL	– Should increase critical nutrient thresholds^(24)^ – Should modify the proposed serving sizes^(24)^ – Should change the wording in warning labels from ‘Excess in’ to ‘High in’ – Should make modifications to the nutrient profile, as it violates the General Health Law^(22)^ – Hinders reformulation^(18,22)^	– The model is evidence-based and tailored to address critical health needs in the region^(19,20,25,26)^	Used the PAHO Nutrient Profile Model as the guiding criteria for the implementation of FOPNL	– Remained unchanged – MoE explanation: PAHO participated in the working groups that reviewed the public comment submissions and presented enough evidence to substantiate the use of this model – (Public health win)
Standardised sizes	Established a standardised serving size of 100 g or 100 ml as reference for all nutrition information on prepackaged products	– Critical nutrients should be listed per ‘typical’ portion size^(17,22)^	– Promotes consumer comprehension and allows for easier comparison of the nutritional value across products^(19–21,26,27)^	Established a standardised serving size of 100 g or 100 ml as reference for all nutrition information on prepackaged products	– Remained unchanged – MoE explanation: allowed in conformity with the Federal Law for Consumer Protection – (Public health win)
Ban on cartoon characters	Banned all cartoon characters and children-targeted advertising on products with FOPNL warning labels	– Violates local and international laws and agreements (e.g. Federal Copyright Law and the World Intellectual Property Organization Copyright Treaty) ^(16,17,22,23)^	– Evidence-based measure, supported by entities like UNICEF and WHO^(19,21,25–27)^ – Does not infringe on any international trade laws) ^(24)^	Banned all cartoon characters and children-targeted advertising on products with FOPNL warning labels	– Remained unchanged – MoE explanation: The use of characters and other advertising elements promotes the consumption of products that pose a risk to the health of children – (Public health win)

NOM, Normal Oficial Mexican (Official Mexican Standards); FOPNL, front-of-pack nutrition labelling; MoE, Ministry of Economy.

Supporting stakeholder groups: Mexican National Institute of Public Health (INSP), Contra Peso, El Poder del Consumidor, the Hunger Project, the Global Health Advocacy Incubator and the World Public Health Nutrition Association.

Opposing stakeholder groups: Business Coordinating Council (CCE), Mead Johnson Nutrition, the Mexican Meat Council, the Confederation of Industrial Chambers of the United Mexican States (CONCAMIN) and the Mexican Council of the Consumer Products Industry (CONMEXICO), which represent some of the largest food and beverage companies in the world including Nestlé, Kellogg, Hershey, Danone, Unilever, Mars, Coca Cola, PepsiCo and Grupo Bimbo.

To analyse the implementation and enforcement of FOPNL in Mexico, we applied Knill and Tosun’s policy cycle model to identify best practices in implementing effective public policies^(28)^. The policy cycle model has been applied with several variations, but in general it contains five stages, including (1) agenda setting, (2) policy formulation, (3) policy adoption, (4) implementation and (5) evaluation^(28)^. For our study, we focus exclusively on the implementation (stage 4) and on some early evaluations (stage 5) published. Within the implementation stage, we examine (a) the Standard (guidelines for implementing the law), (b) monitoring, enforcement and compliance, and (c) barriers and challenges to implementation following previous analyses^(13)^. In doing so, we also document the actions and arguments of supporting and opposing stakeholder positions in each of these areas.

Evaluation represents the final stage of the policy cycle model where often times knowledgeable experts evaluate processes and policy objectives. This stage is key to improve the policy and develop it stronger with progressively improved health outcomes and then restarts the process of policy development. Based on news and advocacy sourced documents outlined in the previous data collection section, we analyse the early stages of evaluation seeking to understand how immediate outcomes were evaluated and the immediate changes observed in relation to potential health benefits.

## Results

### The Official Mexican Standard (October 2019–March 2020)

While the mandate for a FOPNL warning label system was approved through a reform to the General Health Law in October 2019, the new national Standard was concurrently developed. In August 2019, the government established a working group comprised of government officials, health advocates, academics, intergovernmental organisations and the private sector to initially discuss the Standard regulations as described elsewhere^(11)^ and on 8 October 2019, the Ministry of Economy (MoE) and the Federal Commission for the Protection Against Sanitary Risks (COFEPRIS), a decentralised body of the Ministry of Health (MoH), published a draft of the Standard. Following this, the MoE and MoH held a public consultation open for a 2-month period (11 October 2019–10 December 2019) for public commentary, in which supporting and opposing stakeholders provided comments regarding the implementation of the new FOPNL Standard (Table 1). A total of 792 comments were submitted. On 10 March 2020, the MoE and MoH, with assistance from the working group, published a summary of the public comments along with their responses, and the final modification to the Standard was officially published on 27 March 2020. Our summary of the key nine provisions, stakeholder groups and positions and final decisions are presented in Table 1 and described below.

### Key provision #1 (ban on health claims)

A largely contended provision in the draft Standard was a ban on health claims, which prohibited professional health association claims and endorsements on the labels of products with FOPNL warnings. Opposing stakeholders argued the ban violated Article 32 of the Federal Law for Consumer Protection, which establishes minimum requirements for using product endorsements including evidence-based claims^(16,17,29)^. They also insinuated that, since many professional health associations have close ties to the MoH, such a ban would disqualify the MoH’s credibility^(18)^. Supporting stakeholders defended this provision arguing that under the Federal Law for Consumer Protection, all information relating to advertised products must be free of confusing or deceiving elements. In addition, the government would have the right to enforce regulation related to intellectual property based on its legitimate public health interest^(19–21)^. Supporting stakeholders also argued that evidence had shown that some medical associations (e.g. Mexican Diabetes Federation) that offer endorsements have reportedly received funding from industry actors such as Nestlé representing a clear conflict of interest^(19,20)^. Ultimately, the ban was retained in the final Standard as the MoE declared that endorsements and recommendations would only be permitted on products with no FOPNL warnings – in conformity with the Federal Law for Consumer Protection – to promote informed consumption^(24)^ (Table 1).

### Key provision #2 (warning labels for added sweeteners)

The draft Standard also proposed implementing warning labels for added sweeteners (black octagons with white text warning the excess of this critical nutrient proportional to the size of the label’s main display area ranging up to 3·5 × 3·88 cm^2^ for larger products with a display area > 300 cm^2^), which would read ‘Contiene edulcorantes, no recommendable en niños’ (contains sweeteners, not recommended for children). The Standard defined sweeteners as any substance other than monosaccharides and disaccharides that provide a sweet flavor; thus, products containing any synthetic or natural sweeteners, non-caloric sweeteners or polyalcohol would display a FOPNL warning. Opposing stakeholders claimed that widely recognised bodies such as the US Food and Drug Administration (FDA) and CODEX Alimentarius deemed sweeteners as safe^(18,22,23)^. CODEX, an international standard setting entity for nutritional labelling, has close ties to the food and beverage industry^(30)^. Furthermore, they argued it would pose unnecessary obstacles to trade, effectively violating the World Trade Organization (WTO) Technical Barriers to Trade (TBT) Agreement – a common argument used by industry actors to block public health policies^(24)^. Industry actors also suggested that the phrase ‘Avoid in children’ would restrict the consumption of these products, which would be beyond the authority of implementing this regulation^(24)^. Lastly, they argued that this provision would hinder possible reformulation efforts, despite the lack of evidence to support this claim^(23)^. Supporting stakeholders offered evidence surrounding the potential association between sweeteners and NCD and emphasised the importance of implementing visible FOPNL seals, especially since products containing sweeteners are often marketed to children^(19,20)^. In response, the MoE agreed to keep the FOPNL redesigned into a smaller caption (a slim black box with white text) in conformity with Article 212 of the General Health Law Article. The MoE supported that sweeteners are not recommended for children, since they can instil a preference for sweet flavours. Nevertheless, the wording of the warning text was amended to ‘No es recomendable para niños’ (not recommended for children) representing another win for opposing stakeholders.

### Key provision #3 (warning label display areas)

The draft Standard required smaller products with a label display area of ≤ 20 cm^2^ to carry only one simplified warning label with the digit (e.g. 1, 2, 3) corresponding to the number of critical nutrients (e.g. sugar) they contain. Inevitably, these simplified warning labels would be less interpretative for consumers. In response, opposing stakeholders claimed smaller products should not require a warning label as the proposed dimensions would negatively impact the visibility of other mandatory elements on the label^(24)^. Moreover, they argued that products with a main display area < 20 cm^2^ are not legally required to include nutrition facts and should therefore be exempt^(24)^. On the other hand, supporting stakeholders urged policy-makers to establish narrower parameters for smaller products with the label size (≤ 10 cm^2^), given the industry’s previous efforts in Peru to decrease their portion sizes and thereby the size of their packaging in order to evade FOPNL measures^(20,31)^. Although opposing stakeholders were unsuccessful at persuading decision-makers to eliminate this requirement, the MoE doubled the parameter (< 40 cm^2^), allowing larger packages to adopt laxer FOPNL warnings arguing the smaller dimensions would obstruct the warning label visibility in agreement with the industry.

### Key provision #4 (warning labels on multipacks)

The draft Standard required that all products sold in multipacks (e.g. 20 individual packs) display a warning label on each individual pack. Opposing stakeholders maintained that since individual products in multipacks are required to include the label ‘Not for individual sale’, they would not be subject to FOPNL requirements^(22)^. Conversely, supporting stakeholders instead concentrated on advocating for an increase in the label size threshold mentioned in the key provision above^(20)^. The MoE accepted the industry’s comments without explanation and only required that the outer package comply with the FOPNL specifications.

### Key provision #5 (‘product does not contain FOPNL warning labels’ claims)

The draft Standard established that products that did not exceed any of the critical nutrient thresholds would be prohibited from indicating that they were FOPNL-free. Opposing stakeholders requested the elimination of this provision, as they argued that it would violate Article 1 of the Federal Law for Consumer Protection, which calls for clear and accurate information about the content, composition and characteristics of all products^(22,29)^. On the other hand, supporting stakeholders defended this measure, arguing that products should not be able to use these claims as an advertising technique to make products appear healthier than they actually are – commonly referred to as a health halo effect^(24)^. A stakeholder highlighted the importance of upholding this stipulation, based on Peru’s experience, where ultra-processed products such as Cheetos chips use FOPNL-free claims as advertisement^(24)^. Ultimately, the MoE agreed with the industry’s request to eliminate this and grant products without FOPNL warnings the ability to include the text ‘No contiene sellos ni leyendas’ (this product does not contain warning seals or labels) on the main packaging.

### Key provision #6 (placement of warning labels on products with a main display area ≤ 60 cm^2^)

The draft Standard required FOPNL warning labels (octagons) strictly on the upper right corner of their main display area regardless of size allowing consumers to quickly identify products containing an excess of critical nutrients. While some opposing stakeholders falsely claimed a lack of evidence to support the use of FOPNL warning labels altogether, others argued that the placement of these elements would be irrelevant to consumer comprehension^(22,24)^. Moreover, some industry actors argued that given the proposed warning label dimensions, producers should be able to place them anywhere on the product’s main display area^(17)^. On the other hand, based on Chile’s experience, supporting stakeholders emphasised the need to refine this measure by establishing a protected area around the warning labels to prevent producers from including additional, potentially contradictory and messages^(24)^. Despite these recommendations, the MoE agreed with the industry to modify the provision to allow products with a main display area ≤ 60 cm^2^ to display the corresponding FOPNL elements anywhere on their main display area.

### Key provision #7 (Nutrient Profile Model as guiding criteria)

The draft Standard designated the Pan American Health Organization (PAHO) Nutrient Profile Model as the guiding criteria, which establishes critical nutrient cut-offs for FOPNL warning labels. Opposing stakeholders requested policy-makers to modify the nutrient criteria cut-off points^(24)^, modify the standardisation of sizes^(24)^ and change the wording on the FOPNL warning labels from ‘Excess’, which for the industry appears more negative to ‘High in’^(22)^, which can be enable a positive connotation (e.g. high in fibre)^(29)^. If not, opposing stakeholders argued then that the Nutrient Profile Model would have to be modified, claiming it was a violation of the General Health Law and that it would hinder product reformulation^(18,22)^. On the other hand, health advocates expressed their endorsement of this model, due to the ample scientific evidence to support it, and its tailored approach to the health needs of the region, especially fighting against NCD^(19,20,25,26)^. Ultimately, the provision remained unchanged, and the Nutrient Profile Model and the word ‘Excess’ were retained.

### Key provision #8 (standardised portions)

The draft Standard also established a standardised serving size of 100 g or 100 ml as reference for all nutrition information on prepackaged foods and beverages, as recommended by the WHO^(32)^. However, opposing stakeholders argued that critical nutrients should be listed per ‘typical’ portion sizes^(17,22)^, a strategy used to arbitrarily declare smaller portions on the product label than what is commonly consumed by the general population, often resulting in confusion^(33)^. On the contrary, supporting stakeholders applauded this measure as it intended to promote consumer comprehension and allow for easier comparison of the nutritional value across products^(19–21,26,27)^. Despite industry opposition, standardised serving portions remained unchanged, after the MoE stated that this provision was founded on the Federal Law for Consumer Protection.

### Key provision #9 (ban on cartoon characters)

Lastly, the draft Standard put forth a ban on all cartoon characters and children-targeted advertising elements on products with one or more FOPNL warning labels. In response, opposing stakeholders threatened that this ban would violate local and international laws and agreements (e.g. the Federal Copyright Law and the World Intellectual Property Organization Copyright Treaty)^(16,17,22,23)^. Supporting stakeholders expressed their ample support for this evidence-based measure, as they argued that it was endorsed by entities such as UNICEF and the WHO^(19,21,25–27)^. Furthermore, supporting stakeholders in academia provided assistance for the political feasibility of this provision by offering evidence that it would not infringe on any international trade laws (e.g. WTO)^(24)^. In response, the MoE recognised that the use of characters and other advertising elements promote the consumption of products that pose a risk to the health of children, thus securing this ban.

### Implementation of the NOM-051 (the Standard): Phase 1 (October 2020–September 2023)

While the finalised Standard outlined that FOPNL would be rolled out in three phases, this study covers the first phase (October 2020–September 2023). Initially in October 2020, manufacturers were required to place warning labels on the front of packages and from April 2021 the ban on children-targeted advertisement would begin^(34)^.

### Monitoring, enforcement and compliance

In July 2020, the MoE and MoH released a document delineating broadly the criteria for implementing the Standard^(35)^. This agreement appointed the MoE, MoH (COFEPRIS) and the Federal Consumer Protection Agency (PROFECO) as regulatory authorities. In late 2020, PROFECO officials suggested that sanctions for non-compliance with the Standard could range from $2500 USD to nearly $40 000 USD, while deceiving claims on the product packaging could be fined up to $50 000 USD^(36)^.

After 4 months of implementing the Standard during the first phase, in March 2021, the head of PROFECO reported to the media that 99 per cent of the processed food and beverage industry had fully complied with the Standard^(37)^. When asked if they had fined the few companies that had not fully complied, the head of PROFECO reiterated that there had been no need to apply sanctions as the companies had eventually complied with the labelling standard^(37)^.

**Fig. 2:**
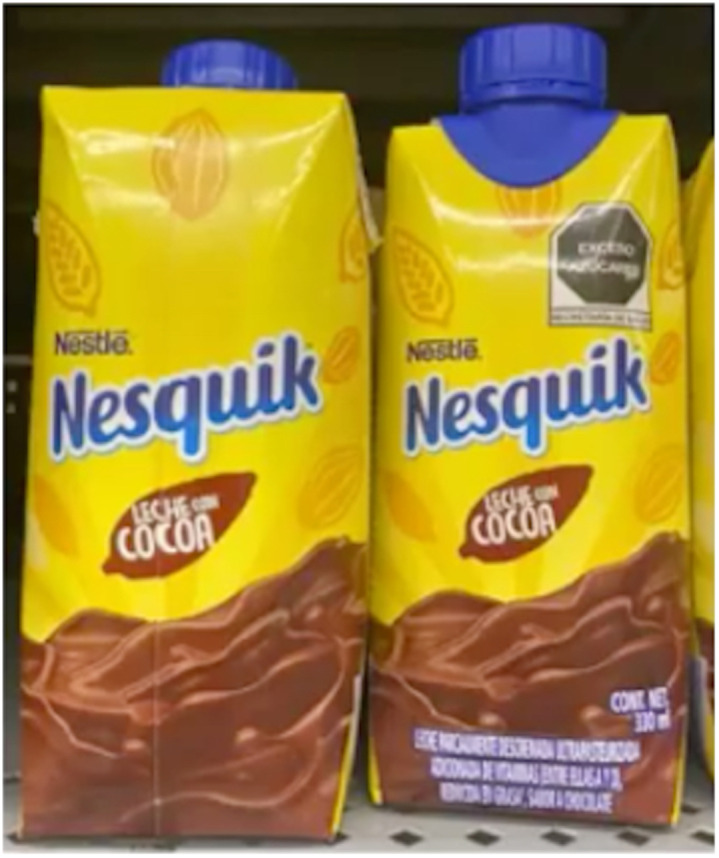
Example of the food and beverage industry using double fronts by making the front and back of the product packaging identical with only one side carrying the warning labels

In the interest of monitoring compliance independently, the civil society organisation El Poder del Consumidor conducted a study between March and May 2021 where they monitored the implementation of FOPNL in ten grocery stores in three states (Queretaro, Mexico City and Morelos)^(38)^. Their goal was to observe the changes on the labels with respect to the warning labels of critical nutrients and the display of cartoon characters by comparing photographs of food products before and after the Standard was implemented^(38)^. They collected information on thirty-five milk products, seventy-three cereal boxes, and sixty-two sugar-sweetened beverages (excluding milk) and overall concluded FOPNL implementation was successful and the usage of cartoon characters on unhealthy products had decreased. However, they documented that the industry had created ‘double fronts’ by making the front and back of the product packaging identical with only one side carrying the warning labels. They recommended grocery stores implement monitoring measures to identify double fronts so that products with warning labels are showcased properly and not deceive consumers^(38)^ (Fig. 2).

In June 2021, the MoE and MoH (COFEPRIS) and PROFECO published a manual to provide transparency and standardise the criteria surrounding the new specifications of labelling (e.g. ingredient lists, nutritional information and labelling examples) for enforcement^(39)^. In July 2021, PROFECO and COFEPRIS officials met with Confederation of Industrial Chambers of the United Mexican States (CONCAMIN) executives nine times to discuss manuals, guides and technical sessions to assist with properly implementing FOPNL^(40)^. As a result, on 26 July 2021, PROFECO and COFEPRIS officials held a technical session workshop open to the public, which summarised a detailed draft of the new specifications and fielded questions^(40)^. When asked about products being displayed incorrectly on shelves, government officials suggested it was up to vendors to enforce the correct display of products at the point of sale, while avoiding the topic of verifications or sanctions^(40)^. Following the lack of information presented during the session, a couple days later the MoE’s director took more of a cooperative rather than confrontational approach as he assured the press that the government did not seek to sanction companies but rather work with them to ensure the labelling was complied with.

In late 2021 and early 2022, the media also reported that government agencies were further monitoring the second stage (banning cartoon characters) of implementation. As of November 2021, at least fifty product and retailer brands – such as Coca-Cola FEMSA, Grupo Bimbo, and Nestlé – had been found possibly non-compliant with the Standard, yet it remains unclear whether COFEPRIS or PROFECO formally issued sanctions^(41)^. Nevertheless, in January 2022, COFEPRIS and PROFECO carried out two raids, during verification visits across the nation to supervise non-compliance by Kellogg Company Mexico^(42)^. During the first raid, over 9000 products that did not display the appropriate warning seals and labels were confiscated at 75 points of sale^(42)^. The second raid was carried out at a Kellogg’s distribution centre in Queretaro state, where over 370 000 products were confiscated^(42)^. However, it is still unclear what was the final result of these raids.

### Barriers and challenges to implementation

One of the main challenges to implement the Standard has been industry pushback to delay the implementation of the Standard. Initially, the food and beverage industry attempted to block and delay the Standard by filing injunctions (amparos). In October 2020, ConMéxico claimed that on behalf of thirty companies, including key opposing vocal food and beverage corporations Grupo Bimbo and Coca Cola, it was filing an injunction against the government claiming that there were violations to right to information, health and the overall process conducted for the modification of the Standard^(43)^. As the banning of cartoon characters was set to go into effect in April 2021, ConMéxico again filed an injunction claiming that this would violate advertising freedoms of expression and intellectual property^(44)^. In April 2021, the MoE claimed that fifty injunctions against the Standard had been filed. While the injunctions have not been publicly disclosed and appear to remain pending, the MoE and MoH continued to implement and enforce the Standard^(44)^.

However, despite the pending injunctions, the industry was still able to delay the implementation and enforcement of the Standard. Initially, the first phase was supposed to begin in October 2020 but due to complaints by the industry that there was a short 6 months (April 2020–September 2020) to comply with the new regulations, especially during COVID-19, enforcement of the Standard was pushed back 2 months until 1 December 2020^(43)^. Secondly, the banning of cartoon characters was originally set to begin on 1 April 2021 but was again delayed 2 months. On 11 March 2021, the National Regulatory Improvement Commission (CONAMER) published an interinstitutional agreement in an effort to establish a 2-month grace period (1 April–31 May 2021) during which producers, importers or traders would not be sanctioned for still advertising cartoon characters on their products^(45)^. The agreement was also distributed to the US Department of Agriculture (USDA) Foreign Agricultural Service and the Global Agricultural Information Network (GAIN) to support the grace period. Eventually the grace period of 2 months was granted as the second stage did not enter into force until 1 June 2021.

**Fig. 3:**
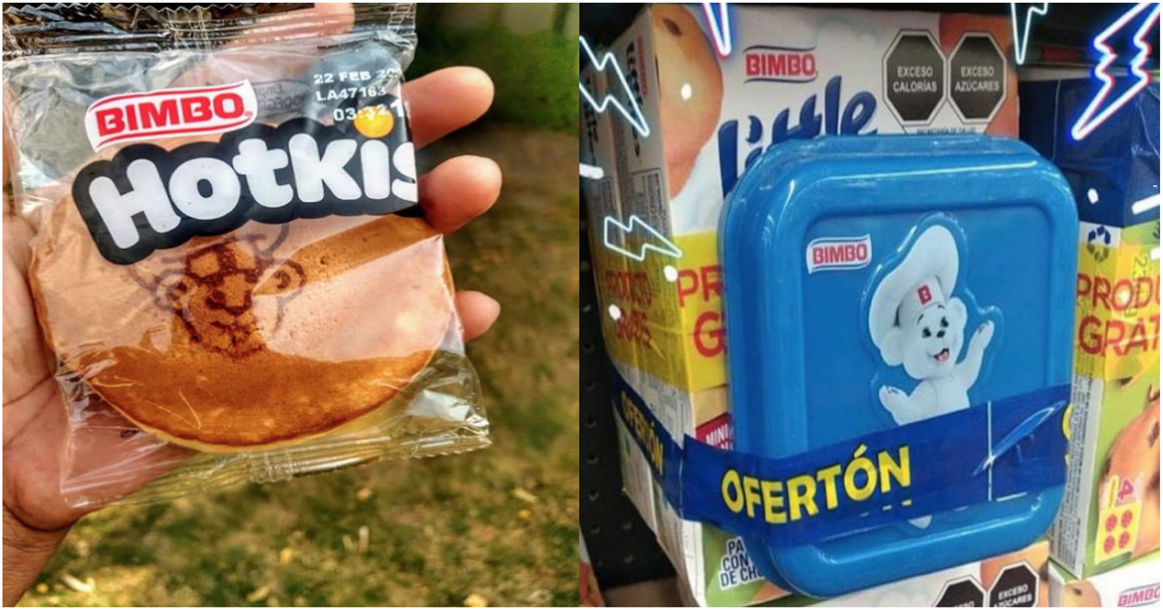
Example of Grupo Bimbo putting their mascot bear as a seal on the product itself or on a promotional container attached to the package

It appears that the biggest challenge to implementing FOPNL has been the industry’s ability to exploit the Standard´s loopholes in regard to implementing FOPNL. Since the beginning of implementation, the application of double fronting on packaged foods has prevented consumers from seeing some of the warning labels. Another loophole is that some companies have found ways to promote their brands’ cartoon characters on the package despite the warning label requirement. For example, Grupo Bimbo put their mascot bear as a seal on the product itself (e.g. pancake) visible through a transparent wrapper rather than on the packaging or on a promotional container attached to the package^(46)^ (Fig. 3). Grupo Bimbo also made a partnership with Pétalo, a hygiene and cleaning company, where they put both Bimbo and Pétalo’s mascot on napkins^(46)^. Media reported that this product generated a boom in sales as many consumers used memes to say ‘Soy inevitable’ (I am unavoidable), which can also be found on social media at #ositoBimbo^(46)^.

Another challenge is that food and beverage companies have found ways to promote their brands’ cartoon characters on other mediums than the package. In response to the ban on cartoon characters, Kellogg announced that El Tigre Toño (Tony the Tiger), Melvin and Sam would continue to live on social media (e.g. Instagram and Twitter)^(47)^. On 4 May 2021, the Kellogg’s characters appeared on Twitter and users complained to the government, but PROFECO responded by stating that the ban of characters was only applicable to the product packaging and asked that these complaints be sent to Kellogg^(47)^. The company also used its Instagram account (@AmigosKelloggs) to tell users to be on the lookout for these characters, even starting the hashtag #SiempreCercadeTi (Always Near You) on the same social media platform^(47)^. Two months later, Kellogg’s hosted a drone light show in Mexico City, where its most popular characters and the phrase ‘Always Near You’ were displayed for almost half an hour. This event was also livestreamed on their Instagram account. This continues to be advertised and as of 1 August 2022 has accumulated nearly 10 000 followers^(47)^.

Food and beverage companies are also seeking to influence purchasing decisions by attempting to bypass warning labels and provide their own information on food while promoting their brands. In February 2021, media reported that companies such as Grupo Bimbo, Lala, Mondelez, Pinsa, among others, have implemented a new barcode functionality in their packaging, where the consumer, scanning with their cell phone, can expand on the information about the products via the app InfoCode^(48)^. InfoCode has more than 200 000 products, and eight out of ten users follow a specific brand for information representing another loophole where companies can promote their brands’ cartoon characters^(48)^.

### Evaluations

To date, there have only been a few evaluations conducted on FOPNL in Mexico. Immediately following the initial implementation of FOPNL, the newspaper *Reforma* conducted a phone survey between 10/9/20 to 10/12/20, in which they asked 400 adults about their thoughts regarding the new FOPNL^(49)^. Although food companies were granted a grace period to implement the new FOPNL by 1 December 2020, half of the adults surveyed said they better understood the nutritional information of products based on the FOPNL, while 31 % did not know or did not answer and 19 % said it did not make nutritional information easier to understand^(49)^. Furthermore, almost half of survey participants said FOPNL would help reduce childhood obesity^(49)^.

In June 2021, the INSP announced it would design a cohort study that evaluates participants’ diets and the type of foods they consumed and their quality before and after the implementation of FOPNL. The study also aims to examine changes in the perception of healthiness and preference of products due to warnings. They are currently conducting this study, and the results are expected to be published in 2023.

On 1 August 2022, the National Health and Nutrition Survey (ENSANUT) 2021 conducted by the National Institute of Public Health (INSP) was presented by the Health Ministry. Among other public health topics, ENSANUT 2021 integrated a questionnaire to evaluate the approval, understanding, use and changes in food purchases after the implementation of FOPNL. The results showed that warning labelling system was the element most consulted by consumers for product information (66·7 %), and a large part of respondents (82·3 %) could correctly identify that a soda drink contained excess sugars. Furthermore, 87·5 % of respondents recognised the presence of critical nutrients on soda products that could cause health damage and 79·2 % responded that they would not give products with warning labels to a child. The population’s perception of the measure was also evaluated, resulting in a national level of ‘good/very good’ (74 %) to identify the excess of calories, critical nutrients or the presence of additives associated with health damage in packaged foods and bottled beverages. Finally, parents were asked about the type of labelling that would help them choose a healthier packaged food and/or bottled beverage for their children, and 60·5 % responded that the FOPNL warning labelling system was the best option. Regarding the changes in the food purchases after FOPNL implementation, 88 % of the parents reported making changes. Of this group, 63 % reduced their purchases of products with warning labels, and 25 % completely stopped buying these products.

## Discussion

The implementation of FOPNL in Mexico appears to be off to a strong start despite attempts by the food and beverage industry and its allies to weaken and delay efforts. During the first phase of implementation, companies are complying with the regulations, removing cartoon characters and reformulating their products to decrease the number of logos their products carry. Process evaluation studies in Mexico are expected in 2023, but an initial survey conducted by civil society showed favourable product compliance. However, future studies should focus on the impact of the FOPNL warning labels on consumer perceptions and awareness such as in Uruguay where an online evaluation study found high levels of awareness, self-reported use of warning labels and the ability to identify products containing excessive amounts of critical nutrients^(4)^. Furthermore, long-term evaluations of the FOPNL from Chile indicate the warning labels have effectively reduced sales of products high in calories, sugars, Na and saturated fats, benefited populations equally across different socio-economic groups and have not negatively impacted the economy^(50)^, despite continued industry claims arguing otherwise.

Given the industry’s relentless attempts to undermine implementation and the lack of resources, political will and state capacity^(13)^, it is imperative that public health groups strengthen their efforts during the drafting of a law’s regulations to minimise industry pressure and ensure high compliance and successful implementation^(13)^. This became true in Mexico as supporting stakeholder groups, local and abroad, appear to have influenced decision-makers to maintain several key provisions for FOPNL, including adhering to PAHO’s Nutrient Profile Model, ensuring standardised size portions, and banning cartoon characters by providing scientific evidence and sharing lessons learned from other countries such as Chile and Peru. Similar efforts have been extended to implementing FOPNL in Uruguay and Argentina^(4)^, and more efforts to improve alliances with other advocacy groups transnationally should continue for other governments attempting to approve and implement FOPNL such as Brazil and Bolivia.

Despite this success, the food and beverage industry was still able to influence decision-makers to weaken several provisions including allowing health claims from industry-backed medical groups, weakening warning label displays, multipacks, warnings for sweeteners, among others. These weakened changes potentially had spillover effects in terms of implementation as the industry has exploited loopholes in the regulations to continue marketing and advertising their cartoon characters in other mediums and designing products with double fronts to deceive consumers at the point of sale. To prevent a similar pattern, governments should prohibit companies from using double fronts and implement complementary food retail environments regulation, especially on point of sale in store and supermarkets as well as related marketing strategies, thereby eliminating loopholes and forcing producers to comply with the regulations and not relying on store owners. Given the industry’s ability to continue marketing their brands’ cartoon characters and products aimed at children on other mediums such as digital spaces, robust regulations need to be strengthen to reduce the power and exposure to marketing.

Food and beverage companies have also attempted to threaten and legally challenge FOPNL in Mexico. Similar to the experience in tobacco control, these represent industry intimidation tactics in attempts to block, weaken and delay public health regulations, especially in low- and middle-income countries^(10,12)^. Other governments may be able to learn from Mexico’s experience that these threats may not result in overturning the regulations as implemented.

### Limitations

Given there were 792 submissions of public comments, this study does not conduct a complete analysis of the public comments limiting the depth of the issues raised. However, one of the study’s strengths is capturing the breadth of information related to implementing FOPNL including enforcement, monitoring, barriers and how the Standard stayed the same and changed based on a summary of the public comments. Also, this study is a descriptive account of the process that did not examine the discourse or different interests within government.

### Conclusion

Early success in implementing FOPNL in Mexico was the result of an inclusive and participatory regulatory process dedicated to maintaining public health advances, local and international health advocacy support, and continued monitoring. Other countries proposing and enacting FOPNL could learn from the Mexican experience to maintain scientifically proven best practices, counter industry barriers and minimise delays in implementation.

## Supplementary material

For supplementary material accompanying this paper visit https://doi.org/10.1017/S1368980023001441. click here to view supplementary material
