# Supplementary material for: Implementing front-of-pack nutrition warning labels in Mexico: important lessons for low- and middle-income countries
Source: Public Health Nutr. 2023 Jul 31;26(10):2149–61. doi: 10.1017/S1368980023001441 (PMC10564596; doi:10.1017/S1368980023001441)
Supplement: Supplementary file 1 [file S1368980023001441sup001.pdf]

Implementación del etiquetado frontal de advertencia en México: Lecciones importantes para países de ingresos bajos y medianos

Eric Crosbie[1,2], Margarita Gabriela Otero Alvarez[1], Michelle Cao[1], Lesly Samara Vejar Renteria[3], Estefania Rodriguez[3], Ana Larrañaga Flota[4] and Angela Carriedo[5,6]

[1]School of Public Health, University of Nevada Reno, Reno, NV 89557, USA: [2]Ozmen Institute for Global Studies, University of Nevada Reno, Reno, NV, USA: [3]Instituto Nacional de Salud Pública, Mexico City, Mexico: [4]University of Sussex, Brighton, UK: [5]Department of Health, University of Bath, Bath, UK: [6]World Public Health Nutrition Association, London, UK

RESUMEN

OBJETIVO: Analizar la implementación del etiquetado frontal nutricional (EFN) en México.
DISEÑO: Revisión de documentos de acceso público, incluidos sitios web legislativos, fuentes de noticias e informes gubernamentales, intergubernamentales y de abogacía. Utilización del modelo de ciclo de políticas para analizar las etapas de implementación y evaluación de la Ley General de Salud de México, modificada con EFN (2019-2022).
RESULTADOS: En octubre de 2019, el gobierno publicó un proyecto de modificación de la Norma Oficial Mexicana para regular y hacer cumplir un nuevo sistema de etiquetado de advertencia EFN. Siguió un período de consulta pública de 60 días (octubre-diciembre de 2019) y la regulación se publicó en marzo de 2020, mientras que la implementación comenzó en octubre de 2020. Un análisis de nueve disposiciones clave de la Norma reveló que la industria de alimentos y bebidas y sus aliados debilitaron algunas disposiciones originales, incluidas declaraciones de propiedades saludables, advertencias sobre edulcorantes agregados y áreas de exhibición. Por otro lado, los grupos de salud pública locales e internacionales defendieron regulaciones clave, incluida la prohibición de publicidad con personajes infantiles, el uso de porciones estandarizadas y criterios de nutrientes siguiendo las mejores prácticas internacionales. La implementación inicial pareció tener un alto cumplimiento y ha contribuido a la reformulación de productos poco saludables. Las barreras continuas para la implementación incluyen los esfuerzos de la industria para crear doble caras frontales y comercializar sus personajes de dibujos animados en las redes sociales y mediante estrategias de marketing digitalizado.
CONCLUSION: El éxito inicial en la implementación del nuevo sistema EFN en México fue el resultado de un proceso regulatorio inclusivo y participativo dedicado a mantener los avances en salud pública, el apoyo a la defensa de la salud local e internacional y el monitoreo continuo. Otros países que propongan y promulguen EFN deberían aprender de la experiencia mexicana con el fin de mantener las mejores prácticas científicamente probadas, contrarrestar las barreras de la industria y minimizar retrasos en la implementación.

INTRODUCCION

La dieta malsana es una de las principales causas de enfermedades no transmisibles (ENT), incluyendo la diabetes tipo 2, enfermedades cardiovasculares y el cáncer, las cuales contribuyen a la mortalidad y la morbilidad en todo el mundo.[1] A nivel mundial, el consumo excesivo de alimentos y bebidas ultraprocesados ha desempeñado un papel importante en el aumento del sobrepeso/obesidad y las ENT, así como las enfermedades relacionadas con la nutrición .[1] Para abordar estos retos, la Organización Mundial de la Salud (OMS) recomienda la implementación de sistemas de etiquetado frontal nutricional efectivos y claros, que

presenten información nutricional en el frente de los alimentos y bebidas envasados que ayude a las poblaciones a comprender el contenido nutricional con el fin de tomar decisiones más saludables, reducir el consumo de alimentos y bebidas ultraprocesados, y que pueda generar una reformulación de alimentos.[2]

Durante la última década, las políticas de EFN han comenzado a expandirse rápidamente en todo el mundo, especialmente en América Latina.[3] Esto comenzó en Chile (2012), seguido de Perú (2013), México (2014), Ecuador (2014), Bolivia (2017), Uruguay (2019), un EFN rediseñado en México (2020), Brasil (2020), Colombia (2021) y Argentina (2021).[3] Otros países de la región están considerando la adopción de un EFN obligatorio para ayudar a abordar la epidemia de ENT en la región.

Muchas de estas políticas obligatorias de EFN han sido obstaculizadas o retrasadas por la industria alimentaria a través de tribunales nacionales e internacionales.[4-6] Al aprender de estas experiencias, los gobiernos son más conscientes y están más dispuestos a proteger los espacios políticos y seguir las recomendaciones clave de organizaciones intergubernamentales como la OMS y los defensores de la salud.[3] El seguimiento de este proceso no solo revela el ritmo de adopción e implementación de políticas[3] sino que subraya cómo la actividad política corporativa puede influir en el proceso político y socavar las políticas de salud pública destinadas a reducir la epidemia de ENT.

En ningún lugar es más importante la situación de las ENT y las enfermedades relacionadas con la nutrición que en México, donde la prevalencia de la obesidad aumentó casi un 50 % entre 2000 y 2018 y las tasas de obesidad severa casi se duplicaron.[7] De particular preocupación es el impacto del sobrepeso/obesidad infantil, el cual afecta a casi cuatro de cada 10 niños en el país.[7] En respuesta, el gobierno mexicano ha implementado varias políticas para abordar este reto, incluido el aumento de los impuestos sobre las bebidas azucaradas y la restricción de la publicidad de alimentos.[8] En octubre de 2019, el gobierno mexicano aprobó una modificación a la Ley General de Salud para establecer un sistema de advertencia EFN que consiste de sellos octagonales de advertencia colocados en productos con alto contenido de azúcar, grasas saturadas, grasas trans, calorías y sal, así como dos leyendas de advertencia para la cafeína y edulcorantes no calóricos en el frente de los paquetes de alimentos y bebidas (Figura 1).

Si bien el proceso legislativo para promulgar políticas de salud pública se ha estudiado exhaustivamente,[9, 10] incluida la aprobación de EFN en México,[11] la etapa de implementación, la cual implica hacer cumplir y evaluar la ley, se ha pasado por alto y no se ha estudiado con la misma importancia, especialmente en países de ingresos bajos y medianos (LMIC, por sus siglas en inglés).[12, 13] Este estudio tiene como objetivo llenar este vacío al examinar las primeras etapas de implementación de EFN en México así como las evaluaciones y resultados emergentes. En particular, examina lo siguiente, a) la regulación (directrices para la implementación de la ley), b) el monitoreo, la aplicación y el cumplimiento, c) las barreras y desafíos para la implementación, y d) la evaluación de la política. A través de esto, el estudio busca brindar lecciones importantes sobre las estrategias de implementación y los desafíos en la implementación para otros LMIC que

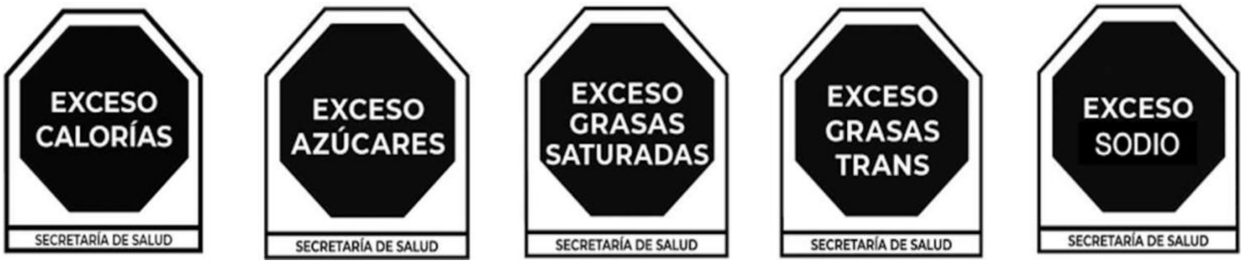

Figura 1: Ejemplo de etiquetado de advertencia en México.

actualmente están proponiendo e implementando EFN.

METODOS

Recopilación de datos

Entre noviembre de 2021 y mayo de 2022, revisamos documentos de acceso público disponibles en Google, incluidos informes de medios de comunicación, informes gubernamentales e intergubernamentales, presentaciones públicas de las partes interesadas clave y sitios web de los poderes legislativos y ejecutivos en México. Se realizaron métodos de búsqueda de bola de nieve estándar en inglés y español, comenzando con términos de búsqueda clave, incluidos "México", "etiquetado", "etiquetado de advertencia", "industria de bebidas" y grupos de partes interesadas específicos de apoyo y oposición. Se localizaron y utilizaron un total de 34 documentos relevantes para este análisis.

Análisis de datos

Para identificar las similitudes y los cambios clave que ocurrieron entre la aprobación y la implementación de EFN en México, primero comparamos el proyecto de norma en Octubre de 2019[14] (antes de la consulta pública) con la Norma Oficial Mexicana (después de la consulta), en lo sucesivo denominada Norma, la cual representa los cambios técnicos obligatorios necesarios para implementar y hacer cumplir la ley, la cual fue publicada en Marzo de 2020.[15]

Después revisamos el resumen del poder ejecutivo con los comentarios públicos sobre el proyecto de modificación a la Norma NOM-051 (la Norma). El resumen abarcó un total de 117 disposiciones, de las cuales analizamos nueve disposiciones clave (pautas, directivas y reglas "dentro" de las leyes y políticas) sobre EFN con base en un requisito mínimo de cinco páginas de resumen. Se excluyeron las disposiciones que no estaban directamente relacionadas con EFN o que no contenían una discusión significativa. A partir de estas nueve disposiciones, identificamos y revisamos los resúmenes del poder ejecutivo así como los comentarios presentados por ocho grupos de partes interesadas que apoyan (defensores de la salud pública y aliados) y ocho que se oponen al EFN (industria de alimentos y bebidas y aliados) (Tabla 1).

Para analizar la implementación y cumplimiento de EFN en México, aplicamos el modelo de ciclo de políticas de Knill y Tosun para identificar las mejores prácticas en la implementación de políticas públicas efectivas.[16] El modelo de ciclo de políticas se ha aplicado con varias variaciones, pero en general contiene cinco etapas, que incluyen 1) establecimiento de la agenda, 2) formulación de políticas, 3) adopción de políticas, 4) implementación y 5) evaluación.[16]

Para nuestro estudio nos enfocamos exclusivamente en la implementación (etapa

4) y en algunas evaluaciones iniciales (etapa 5) publicadas. Para la etapa de implementación, examinamos a) la Norma (directrices para implementar la ley), b) el monitoreo, la aplicación y el cumplimiento y c) las barreras y desafíos para la implementación en línea con análisis previos.[13] De igual manera, documentamos las acciones y los argumentos de apoyo y oposición de las partes interesadas en cada una de estas áreas.

La evaluación representa la etapa final del modelo de ciclo de políticas donde, a menudo, expertos evalúan los procesos y los objetivos de las políticas. Esta etapa es clave para mejorar la política y fortalecerla con resultados de salud progresivamente mejorados para luego reiniciar el proceso de desarrollo de la política. Con base en documentos de fuentes de noticias y abogacía descritos en la sección anterior de recopilación de datos, analizamos las primeras etapas de la evaluación para comprender cómo se evaluaron los resultados y cambios inmediatos observados en relación con los posibles beneficios para la salud.

RESULTADOS

La Norma Oficial Mexicana (octubre de 2019-marzo 2020)

Si bien el mandato para el sistema de etiquetado de advertencia EFN se aprobó a través de una modificación a la Ley General de Salud en octubre de 2019, la nueva Norma oficial se desarrolló de manera simultánea. En agosto de 2019, el gobierno estableció un grupo de trabajo compuesto por funcionarios gubernamentales, defensores de la salud, académicos, organizaciones intergubernamentales y el sector privado para discutir de manera inicial las regulaciones de la Norma como se describe en otros documentos[11] y el 8 de octubre de 2019, la Secretaría de Economía (SE) y la Comisión Federal para la Protección contra Riesgos Sanitarios (COFEPRIS), un organismo descentralizado de la Secretaría de Salud (SS), publicaron un proyecto de la Norma. Después de esto, la Secretaría de Economía y la Secretaría de Salud realizaron una consulta pública abierta por un período de dos meses (11 de octubre de 2019-10 de diciembre de 2019) para comentarios públicos, en la que las partes interesadas de apoyo y oposición proporcionaron comentarios sobre la implementación de la nueva Norma (Tabla 1). Se presentaron un total de 792 comentarios. El 10 de marzo de 2020, la Secretaría de Economía y la Secretaría de Salud, con la ayuda del grupo de trabajo, publicaron un resumen de los comentarios públicos junto con sus respuestas y la modificación final de. La Norma se publicó oficialmente el 27 de marzo de 2020. Nuestro resumen de las nueve disposiciones clave, los grupos de partes interesadas y las posiciones y las decisiones finales se presentan en la Tabla 1 y se describen a continuación.

Disposición clave #1 (prohibición de declaraciones de propiedades saludables)

Una disposición muy discutida en el proyecto de la Norma fue la prohibición de las declaraciones de propiedades saludables, la cual prohibía las declaraciones por parte de asociaciones profesionales de la salud y el uso de avales en las etiquetas de los productos con advertencias EFN. Las partes opuestas argumentaron que la prohibición violaba el artículo 32 de la Ley Federal de Protección al Consumidor, que establece los requisitos mínimos para el uso de avales en productos, incluidas las declaraciones basadas en evidencia científica.[17-19] También insinuaron que, dado que muchas asociaciones profesionales de salud tienen vínculos estrechos con la Secretaría de Salud, dicha prohibición descalificaría la credibilidad de la Secretaría de Salud.[20] Las partes interesadas que defendieron esta disposición argumentaron que, según la Ley Federal de

Protección al Consumidor, toda la información relacionada con los productos publicitados debe estar libre de elementos confusos o engañosos. Además, el gobierno tendría derecho a hacer cumplir la regulación relacionada con la propiedad intelectual en función de su interés legítimo por la salud pública.[21-23] Las partes interesadas respaldando esta disposición también argumentaron que la evidencia había demostrado que algunas asociaciones médicas (p. ej., la Federación Mexicana de Diabetes) que avalan productos han recibido financiamiento de actores de la industria como Nestlé, lo que representa un claro conflicto de interés. [21, 22] Al final, se mantuvo la prohibición en la versión final de la Norma ya que la Secretaría de Economía declaró que el uso de avales y recomendaciones solo se permitiría en productos sin el etiquetado de advertencia–de conformidad con la Ley Federal de Protección al Consumidor– con el fin de promover el consumo informado [24] (Tabla 1).

Disposición clave #2 (sellos de advertencia para edulcorantes* agregados*)

El proyecto de la Norma también propuso implementar sellos de advertencia para edulcorantes agregados (octágonos negros con texto blanco advirtiendo el exceso de este nutriente crítico proporcional al tamaño del área de exhibición principal de la etiqueta, que llegan hasta los 3,5x3,88 cm2 para productos más grandes con un área de exhibición > 300 cm2), los cuales dirían "Contiene edulcorantes, evitar en niños." La Norma definió los edulcorantes como cualquier sustancia distinta de los monosacáridos y disacáridos que proporciona un sabor dulce, por lo tanto, los productos que contienen edulcorantes sintéticos o naturales, edulcorantes no calóricos o polialcohol mostrarían una advertencia EFN. Las partes interesadas en oposición afirmaron que organismos ampliamente reconocidos, como la Administración de Alimentos y Medicamentos de Estados Unidos (FDA, por sus siglas en inglés) y CODEX Alimentarius, consideraban que los edulcorantes son seguros.[20, 25, 26] CODEX, un organismo internacional que establece estándares para el etiquetado nutricional, tiene estrechos vínculos con la industria de alimentos y bebidas.[27] Además, argumentaron que plantearía obstáculos innecesarios para el comercio, violando así el Acuerdo de Obstáculos Técnicos al Comercio (OTC) de la Organización Mundial del Comercio (OMC), un argumento comúnmente utilizado por los actores de la industria para bloquear las políticas de salud pública.[24] Los actores de la industria también argumentaron que la frase "Evitar en niños" restringiría el consumo de estos productos, lo cual sobrepasaría la autoridad para implementar esta regulación.[24] Por último, argumentaron que esta disposición obstaculizaría los posibles esfuerzos de reformulación, a pesar de la falta de evidencia para respaldar este razonamiento.[26] Las partes interesadas que apoyaron el proyecto ofrecieron evidencia sobre la posible asociación entre los edulcorantes y las ENT y enfatizaron la importancia de implementar sellos de advertencia EFN visibles, especialmente porque los productos que contienen edulcorantes a menudo se promocionan a los niños. [21, 22] En respuesta, la Secretaría de Economía acordó mantener el EFN pero rediseñado en una leyenda más pequeña (un recuadro negro delgado con texto blanco) de conformidad con el artículo 212 de la Ley General de Salud. La Secretaría de Economía concordó que los edulcorantes no se recomiendan para los niños, ya que pueden inculcar una preferencia por los sabores dulces. Sin embargo, la redacción del texto de advertencia se modificó a “No recomendable en niños,” lo que representa otra victoria para las partes interesadas en oposición.

Disposición clave #3 (área de exhibición del etiquetado de advertencia)

El proyecto de Norma requería que los productos más pequeños con un área de exhibición en la etiqueta de ≤20 cm2 llevaran solo un sello de advertencia simplificado con el dígito (p. ej., 1, 2, 3) correspondiente a la cantidad de nutrientes críticos (p. ej., azúcar) que contienen. Inevitablemente, estos sellos de advertencia simplificados serían menos interpretativos para los consumidores. En respuesta, las partes interesadas en oposición afirmaron que los productos más pequeños no deberían requerir un sello de advertencia, ya que las dimensiones propuestas afectarían negativamente la visibilidad de otros elementos obligatorios en la etiqueta.[24]Además, argumentaron que los productos con un área de exhibición principal <20 cm2 no están legalmente obligados a incluir información nutricional y, por lo tanto, deberían estar exentos.[24] Por el otro lado, las partes interesadas que apoyaban la propuesta instaron a los legisladores a establecer parámetros más estrechos para productos más pequeños, con el tamaño de la etiqueta siendo ≤10 cm2, dados los esfuerzos previos de la industria en Perú para reducir el tamaño de las porciones y, por lo tanto, el tamaño de sus envases para evadir las medidas de EFN.[22, 28] Aunque las partes interesadas opuestas no lograron persuadir a los tomadores de decisión para que eliminaran este requisito, la Secretaría de Economía duplicó el parámetro (<40 cm2), lo que permitió que paquetes más grandes adoptaran advertencias EFN más laxas, argumentando que las dimensiones más pequeñas obstruirían la visibilidad del etiquetado de advertencia, de acuerdo con los argumentos de la industria.

Disposición clave #4 (etiquetado de advertencia en empaques colectivos)

El proyecto de Norma requería que todos los productos comercializados en empaques colectivos (p. ej., 20 paquetes individuales) mostraran un sello advertencia en cada paquete individual. Las partes interesadas que se oponían sostuvieron que, dado que los productos individuales en empaques colectivos deben incluir la etiqueta "No para la venta individual", no estarían sujetos a los requisitos de EFN.[25] Por el contrario, las partes interesadas que apoyaban la disposición se concentraron en abogar por un aumento en los parámetros del tamaño de la etiqueta mencionado en la disposición clave anterior. [22] La Secretaría de Economía aceptó los comentarios de la industria sin explicación y exigió que solo el paquete exterior cumpliera con las especificaciones de EFN.

Disposición clave #5 (declaración libre de EFN ***"este producto no contiene sello ni leyendas")***

El proyecto de Norma establecía que los productos que no excedieran ninguno de los umbrales de nutrientes críticos tendrían prohibido indicar que estaban libres de EFN. Los actores en oposición solicitaron la eliminación de esta disposición, ya que argumentaron que violaría el artículo 1 de la Ley Federal de Protección al Consumidor, que exige información clara y precisa sobre el contenido, la composición y las características de todos los productos. [19, 25] Por otro lado, los actores apoyando el proyecto defendieron esta medida, argumentando que los productos no deberían poder utilizar estas afirmaciones como una técnica publicitaria para hacer que los productos parezcan más saludables de lo que realmente son, lo que comúnmente se conoce como el "efecto halo de salud".[24] Un actor destacó la importancia de mantener esta disposición, con base en la experiencia de Perú, donde los productos ultraprocesados, como las frituras Cheetos, usan la declaración

de libre de EFN como publicidad.[24] Al final, la Secretaría de Economía estuvo de acuerdo con la solicitud de la industria de eliminar esta disposición y otorgar a los productos sin etiquetado de advertencia EFN la capacidad de incluir el texto “No contiene sellos ni leyendas” en el empaque principal.[24]

Disposición clave #6 (posicionamiento del etiquetado de advertencia en productos con área de exhibición principal ≤60cm2)

El proyecto de Norma requería sellos de advertencia EFN (octágonos) estrictamente en la esquina superior derecha del área de exhibición principal, independientemente del tamaño, lo que permitía a los consumidores identificar rápidamente los productos que contenían un exceso de nutrientes críticos. Mientras que algunos actores en oposición alegaron falsamente la falta de evidencia para respaldar el uso del etiquetado de advertencia EFN, otros argumentaron que la ubicación de estos elementos sería irrelevante para la comprensión del consumidor. [24, 25] Además, algunos actores de la industria argumentaron que dadas las dimensiones de los sellos de advertencia propuestas, los productores deberían poder colocarlas en cualquier lugar del área de exhibición principal del producto.[18] Por otro lado, con base en la experiencia de Chile, las partes interesadas apoyando la disposición enfatizaron la necesidad de perfeccionar esta medida mediante el establecimiento de un área protegida alrededor de los sellos de advertencia para evitar que los productores incluyan mensajes adicionales, potencialmente contradictorios. [24] A pesar de estas recomendaciones, la Secretaría de Economía concordó con la industria al modificar la disposición para permitir que los productos con un área principal de exhibición ≤60 cm2 exhiban los elementos EFN correspondientes en cualquier lugar de su área principal de exhibición.

Disposición clave #7 (Modelo de Perfil de Nutrientes como criterio rector)

El proyecto de Norma designó el Modelo de Perfil de Nutrientes de la Organización Panamericana de la Salud (OPS) como el criterio rector, el cual establece límites de nutrientes críticos para el etiquetado de advertencia EFN. Los actores en oposición solicitaron a los legisladores que modificaran los umbrales de los nutrientes, [24] modificaran la estandarización de los tamaños de porción, [24] y cambiaran la redacción en los sellos de advertencia EFN de “Exceso”, el cual a la industria le parece más negativa, a “Alto en” [25], lo que puede conllevar una connotación positiva (p. ej., alto contenido de fibra).[29] Los actores en oposición argumentaron que, de lo contrario, el Modelo de Perfil de Nutrientes tendría que modificarse, alegando que era una violación de la Ley General de Salud y que obstaculizaría la reformulación de los productos.[20, 25] Por otro lado, los defensores de la salud expresaron su respaldo a este modelo, debido a la amplia evidencia científica que lo respalda, y su enfoque adaptado a las necesidades de salud de la región, especialmente la lucha contra las ENT. [21, 22, 30, 31] Al final, la disposición se mantuvo sin cambios y se mantuvo el Modelo de Perfil de Nutrientes y el uso de la palabra "Exceso".

Disposición clave #8 (porciones estandarizadas)

El proyecto de Norma también estableció un tamaño de porción estandarizado de 100 g o 100 ml como referencia para toda la información nutricional en alimentos y bebidas preenvasados, según lo recomendado por la OMS.[32] Sin embargo, los actores en oposición argumentaron que los nutrientes críticos deben declararse por tamaño de porción "típico",[18, 25]una estrategia utilizada

para declarar arbitrariamente porciones más pequeñas en la etiqueta del producto que lo que consume comúnmente la población en general, lo que a menudo genera confusión.[33] Por el contrario, los defensores de la salud y aliados aplaudieron esta medida ya que pretendía promover la comprensión del consumidor y permitir una comparación más fácil del valor nutricional entre productos. [21-23, 29, 31] A pesar de la oposición de la industria, las porciones estandarizadas se mantuvieron sin cambios, después de la Secretaría de Economía declarara que esta disposición se basaba en la Ley Federal de Protección al Consumidor.

Disposición clave #9 (prohibición de personajes animados)

Por último, el proyecto de norma proponía la prohibición de todos los personajes animados y otros elementos publicitarios dirigidos a niños en productos con uno o más sellos de advertencia EFN. En respuesta, los actores en oposición argumentaron que esta prohibición violaría las leyes y los acuerdos nacionales e internacionales (p. ej., la Ley Federal del Derecho de Autor, Tratado de la Organización Mundial de la Propiedad Intelectual sobre Derecho de Autor). [17, 18, 25, 26] Las partes interesadas que secundaron el proyecto expresaron su amplio apoyo a esta medida basada en evidencia, ya que argumentaron que fue respaldada por entidades como UNICEF y la OMS. [21, 23, 29-31] Además, partes interesadas en la academia brindaron asistencia para demostrar la viabilidad política de esta disposición al ofrecer evidencia de que no infringiría ninguna ley de comercio internacional (por ejemplo, la OMC). [24] En respuesta, la Secretaría de Educación reconoció que el uso de personajes y otros elementos publicitarios promueven el consumo de productos que representan un riesgo para la salud de los niños, conservando así esta prohibición.

Implementación de la NOM-051 (la Norma): Fase 1 (octubre 2020-septiembre 2023)

Si bien la versión final de la Norma estableció que el etiquetado frontal se implementaría en tres fases, este estudio cubre la primera fase (octubre de 2020-septiembre de 2023). Inicialmente, en octubre de 2020, los fabricantes debían colocar el etiquetado de advertencia en el frente de los empaques y, a partir de abril de 2021, comenzaría la prohibición de publicidad dirigida a niños. [34]

Monitoreo, aplicación y cumplimiento

En julio de 2020, la Secretaría de Economía y la Secretaría de Salud publicaron un documento que delinea ampliamente los criterios para implementar la Norma. [35] Este acuerdo designó la Secretaría de Economía, la Secretaría de Salud (COFEPRIS) y la Procuraduría Federal del Consumidor (PROFECO) como autoridades reguladoras. A fines de 2020, los funcionarios de PROFECO sugirieron que las sanciones por incumplimiento de la Norma podrían oscilar entre $2500 USD y hasta $40 000 USD, mientras que las declaraciones engañosas en el empaque del producto podrían recibir multas de hasta $50 000 USD. [36]

Después de cuatro meses de implementación de la Norma durante la primera fase, en marzo de 2021, el titular de la PROFECO informó a los medios de comunicación que el 99% de la industria de alimentos y bebidas procesados había cumplido a cabalidad con la Norma. [37] Cuando se le preguntó si habían multado a las pocas empresas que no habían cumplido, el titular de la PROFECO reiteró que no hubo necesidad de aplicar sanciones ya que las empresas finalmente cumplieron con la norma de etiquetado. [37]

Con el interés de monitorear el cumplimiento de manera independiente, la

organización de la sociedad civil El Poder del Consumidor realizó un estudio entre marzo y mayo de 2021 donde monitorearon la implementación del etiquetado frontal en diez supermercados en tres estados (Querétaro, Ciudad de México y Morelos). [38]

Su objetivo fue observar los cambios en los empaques con respecto al etiquetado de advertencia de nutrientes críticos y la exhibición de personajes animados comparando fotografías de productos alimenticios antes y después de la implementación de la Norma. [38] Recolectaron información sobre 35 productos lácteos, 73 cajas de cereal y 62 bebidas azucaradas (excluida la leche) y, en general, concluyeron que la implementación del etiquetado frontal fue exitosa y que el uso de personajes animados en productos no saludables había disminuido. Sin embargo, documentaron que la industria había creado “dobles caras” al hacer que el frente y el dorso del empaque del producto fueran idénticos con solo un lado que exhibe las etiquetas de advertencia. Recomendaron que los supermercados implementen medidas de monitoreo para identificar “dobles caras para que los productos con el etiquetado de advertencia se muestren adecuadamente y no engañen a los consumidores[38] (Figura 2).

En junio de 2021, la Secretaría de Economía, la Secretaría de Salud (COFEPRIS) y la PROFECO publicaron un manual para proporcionar transparencia y estandarizar los criterios en torno a las nuevas especificaciones de etiquetado (p. ej., listas de ingredientes, información nutricional y ejemplos de etiquetado) para su cumplimiento. [39] En julio de 2021, los funcionarios de la PROFECO y la COFEPRIS se reunieron nueve veces con ejecutivos de la Confederación de Cámaras Industriales de los Estados Unidos Mexicanos (CONCAMIN) para discutir manuales, guías y sesiones técnicas para ayudar a implementar adecuadamente el etiquetado frontal. [40] Como resultado, el 26 de julio de 2021, los funcionarios de la PROFECO y la COFEPRIS ofrecieron un taller de sesión técnica abierto al público, donde se resumió un borrador detallado de las nuevas especificaciones y se respondieron preguntas. [40] Cuando se les preguntó sobre los productos que se exhibían incorrectamente en los estantes, los funcionarios del gobierno dijeron que era responsabilidad de los proveedores hacer cumplir la correcta exhibición de los productos en el punto de venta, evitando el tema de las verificaciones o sanciones. [40]Tras la falta de información presentada durante la sesión, un par de días después, el director de la Secretaría de Salud tomó más un enfoque cooperativo que de confrontación, ya que aseguró a la prensa que el gobierno no buscaba sancionar a las empresas, sino trabajar con ellas para garantizar que se cumpliera con el etiquetado.

A fines de 2021 y principios de 2022, los medios de comunicación también informaron que las agencias gubernamentales estaban monitoreando más la segunda etapa (prohibición de personajes animados) de implementación. A noviembre de 2021, al menos 50 marcas de productos y distribuidores, como Coca-Cola FEMSA, Grupo Bimbo y Nestlé, habían sido encontradas incumpliendo con la Norma, pero aún no está claro si COFEPRIS o PROFECO emitieron sanciones de manera formal.[41] Sin embargo, en enero de 2022, COFEPRIS y PROFECO llevaron a cabo dos operativos durante visitas de verificación en todo el país para supervisar el incumplimiento por parte de Kellogg Company México.[42] Durante el primer operativo más de 9, 000 productos que no exhibían los sellos y leyendas de advertencia correspondientes fueron decomisados en 75 puntos de venta.[42] El segundo operativo se llevó a cabo en un centro de distribución de Kellogg's en el estado de Querétaro, donde se

decomisaron más de 370,000 productos.[42] Sin embargo, aún no está claro cuál fue el resultado final de estos operativos.

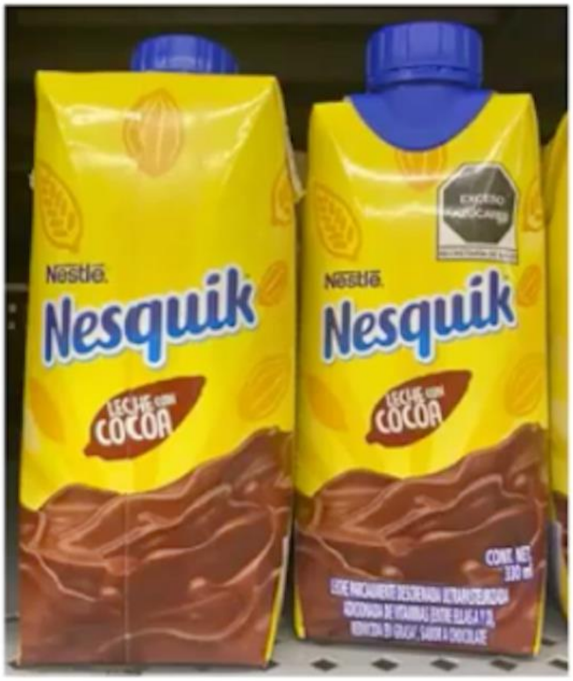

Figura 2: Ejemplo de las doble caras utilizadas por la industria de alimentos y bebidas al hacer que el frente y el reverso del empaque del producto sean idénticos con solo un lado que lleva el etiquetado de advertencia.

Barreras y desafíos para la implementación

Uno de los principales desafíos para implementar la norma ha sido el esfuerzo de la industria para retrasar la implementación de la norma. Inicialmente, la industria de alimentos y bebidas intentó bloquear y retrasar la Norma presentando amparos. En octubre de 2020, ConMéxico afirmó que en nombre de 30 empresas, incluidas las corporaciones de alimentos y bebidas opositoras clave, Grupo Bimbo y Coca Cola, estaba presentando un amparo contra el gobierno alegando que hubo violaciones al derecho a la información, a la salud y al proceso general llevado a cabo para la modificación de la Norma. [43] Dado que la prohibición de los personajes animados entraría en vigencia en abril de 2021, ConMéxico presentó nuevamente un amparo alegando que esto violaría las libertades de expresión publicitarias y la propiedad intelectual.[44] En abril de 2021, la Secretaría de Economía afirmó que se habían presentado 50 amparos contra la Norma. Si bien los amparos no se han divulgado públicamente y parecen permanecer pendientes, la Secretaría de Economía y la Secretaría de Salud continuaron implementando y haciendo cumplir la Norma.[44]

Sin embargo, a pesar de los amparos pendientes, la industria logró retrasar la implementación y el cumplimiento de la Norma. Inicialmente, se suponía que la primera fase comenzaría en octubre de 2020, pero debido a las quejas de la industria de que los seis meses (abril de 2020 a septiembre de 2020) para cumplir con las nuevas regulaciones no serían suficientes, especialmente durante el Covid-19, la aplicación de la norma se retrasó dos meses hasta el 1 de diciembre de 2020.[43] De igual manera, la prohibición de personajes animados originalmente estaba programada para comenzar el 1 de abril de 2021, pero nuevamente se retrasó dos meses. El 11 de marzo de 2021, la Comisión Nacional de Mejora Regulatoria (CONAMER) publicó un acuerdo interinstitucional en un esfuerzo por establecer un período de gracia de dos meses (del 1 de abril al 31 de mayo de 2021) durante el cual los productores, importadores o comerciantes no serían sancionados por seguir publicitando personajes animados en sus productos. [45] El acuerdo también se distribuyó al Servicio de Agricultura Extranjera del Departamento de Agricultura de Estados Unidos (USDA, por sus siglas en inglés) y a la Red Global de Información Agrícola (GAIN, por sus siglas en inglés) para respaldar el período de gracia. Finalmente se concedió el plazo de gracia de

dos meses ya que la segunda etapa no entró en vigor hasta el 1 de junio de 2021.

Parece que el mayor desafío para implementar el etiquetado frontal ha sido la capacidad de la industria para explotar las lagunas de la Norma con respecto a la implementación del etiquetado. Desde el comienzo de la implementación, el uso de doble caras en los alimentos envasados ha impedido que los consumidores vean algunas de las etiquetas de advertencia. Otra laguna es que algunas empresas han encontrado formas de promocionar los personajes de dibujos animados de sus marcas en el empaque a pesar de exhibir sellos de advertencia. Por ejemplo, Grupo Bimbo colocó su oso mascota como un sello en el producto mismo (p. ej., panqueque) visible a través de un envoltorio transparente en lugar de en el empaque o en un contenedor promocional adherido al paquete[46] (Figura 3). Grupo Bimbo también se asoció con Pétalo, una empresa de higiene y limpieza, al colocar la mascota de Bimbo y Pétalo en servilletas.[46] Los medios informaron que este producto generó un incremento en las ventas ya que muchos consumidores usaron memes para decir "soy inevitable", que también se puede encontrar en las redes sociales a través #ositoBimbo.[46]

Otro desafío es que las empresas de alimentos y bebidas han encontrado formas de promocionar los personajes animados de sus marcas en otros medios además del empaque. En respuesta a la prohibición de los personajes animados, Kellogg's anunció que El Tigre Toño, Melvin y Sam continuarían viviendo en las redes sociales (p. ej., Instagram, Twitter).[47] El 4 de mayo de 2021, los personajes de Kellogg's aparecieron en Twitter y los usuarios se quejaron ante el gobierno, pero la PROFECO respondió afirmando que la prohibición de los personajes solo se aplicaba al empaque del producto y pidió que estas quejas se dirijieran a Kellogg's.[47] La empresa también utilizó su cuenta de Instagram (@AmigosKelloggs) para decirles a los usuarios que estuvieran atentos a estos personajes, incluso iniciando el hashtag #SiempreCercadeTi en la misma plataforma de redes sociales.[47] Dos meses después, Kellogg's organizó un espectáculo de luces con drones en la Ciudad de México, donde sus personajes más populares y la frase "Siempre cerca de ti" se exhibieron durante casi media hora. Este evento también fue transmitido en vivo en su cuenta de Instagram. Esto continúa publicitándose y al 1 de agosto de 2022 ha acumulado casi 10,000 seguidores.[47]

Las empresas de alimentos y bebidas también buscan influir en las decisiones de compra al intentar eludir las etiquetas de advertencia y proporcionar su propia información sobre los alimentos al promocionar sus marcas. En febrero de 2021, los medios informaron que empresas como Grupo Bimbo, Lala, Mondelez, Pinsa, entre otras, han implementado una nueva funcionalidad de código de barras en sus empaques, donde el consumidor, al escanear con su celular, puede ampliar la información de los productos a través de la aplicación InfoCode.[48] InfoCode tiene más de 200,000 productos y ocho de cada diez usuarios siguen una marca específica para obtener información, lo que representa otro vacío legal donde las empresas pueden promocionar los personajes animados de sus marcas.[48]

Evaluaciones

Hasta la fecha, solo se han realizado unas pocas evaluaciones sobre el etiquetado frontal en México. Inmediatamente después de la implementación inicial del etiquetado, el periódico Reforma realizó una encuesta telefónica entre el 9/10/20 y el 12/10/20, en la que preguntó a 400 adultos acerca de su

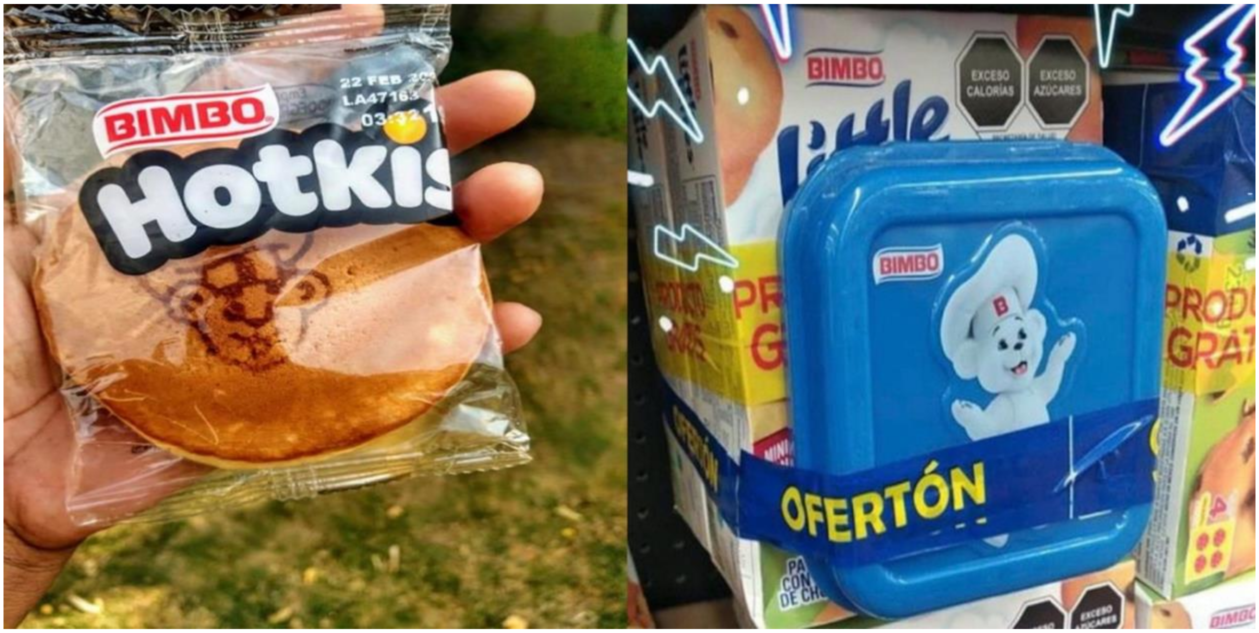

Figura 3: Ejemplo de Grupo Bimbo colocando su oso mascota como sello en el producto mismo o en un envase promocional adjunto al empaque.

opinión sobre el nuevo etiquetado.[49] Aunque a las empresas de alimentos se les otorgó un período de gracia para implementar el nuevo etiquetado antes del 1 de diciembre de 2020, la mitad de los adultos encuestados dijeron que entendían mejor la información nutricional de los productos basados en el etiquetado frontal, mientras que el 31% no sabía o no respondió y el 19% dijo que no facilitaba la comprensión de la información nutricional.[49] Además, casi la mitad de los participantes de la encuesta dijeron que el etiquetado frontal ayudaría a reducir la obesidad infantil.[49]

En junio de 2021, el Instituto Nacional de Salud Pública (INSP), anunció que diseñaría un estudio de cohorte que evaluaría la dieta de los participantes y el tipo de alimentos que consumen, así como su calidad antes y después de la implementación del etiquetado frontal. El estudio también tiene como objetivo examinar los cambios en la percepción de salubridad y preferencia de producto como resultado de las advertencias. Actualmente están realizando este estudio y se espera que los resultados se publiquen en 2023.

El 1 de agosto de 2022, la Secretaría de Salud presentó la Encuesta Nacional de Salud y Nutrición (ENSANUT) 2021 realizada por el INSP. Entre otros temas de salud pública, la ENSANUT 2021 integró un cuestionario para evaluar la aprobación, comprensión, uso y cambios en las compras de alimentos tras la implementación del etiquetado frontal. Los resultados mostraron que el sistema de etiquetado de advertencia fue el elemento más consultado por los consumidores para la información del producto (66.7 %), y una gran parte de los encuestados (82.3 %) pudo identificar correctamente que una bebida gaseosa contenía azúcares en exceso. Además, el 87.5 % de los encuestados reconoció la presencia de nutrientes críticos en las bebidas gaseosas que podrían causar daños a la salud y el 79.2 % respondió que no le darían productos con etiquetas de advertencia a un niño. También se evaluó la percepción de la población sobre la medida, resultando en un nivel nacional de

"bueno/muy bueno" (74%) para identificar el exceso de calorías, nutrientes críticos o la presencia de aditivos asociados con daños a la salud en alimentos y bebidas envasados. Finalmente, se preguntó a los padres sobre el tipo de etiquetado que les ayudaría a elegir un alimento y/o bebida envasado más saludable para sus hijos, y el 60.5% respondió que el sistema de etiquetado de advertencia EFN era la mejor opción. En cuanto a los cambios en las compras de alimentos después de la implementación del etiquetado frontal, el 88% de los padres informaron haber realizado cambios. De este grupo, el 63% redujo sus compras de productos con etiquetado de advertencia y el 25% dejó de comprar estos productos por completo.

DISCUSION

La implementación del etiquetado frontal en México parece tener un buen comienzo a pesar de los intentos de la industria de alimentos y bebidas y sus aliados de debilitar y retrasar los esfuerzos. Durante la primera fase de implementación, las empresas están cumpliendo con las regulaciones, eliminando los personajes animados y reformulando sus productos para disminuir la cantidad de sellos de advertencia que llevan sus productos. Se espera que los estudios de evaluación del proceso en México estén listos en 2023, pero una encuesta inicial realizada por la sociedad civil mostró un cumplimiento favorable del producto. Sin embargo, futuros estudios deben centrarse en el impacto del etiquetado de advertencia EFN en las percepciones y la concientización de los consumidores, como en Uruguay, donde un estudio de evaluación en línea encontró altos niveles de concientización, uso auto informado del etiquetado de advertencia y la capacidad de identificar productos que contienen cantidades excesivas de nutrientes críticos. [4] Además, las evaluaciones a largo plazo del etiquetado frontal en Chile indican que el etiquetado de advertencia ha reducido efectivamente las ventas de productos con alto contenido de calorías, azúcares, sodio y grasas saturadas, ha beneficiado a las poblaciones por igual en diferentes grupos socioeconómicos y no ha tenido un impacto negativo en la economía,[50] a pesar de la industria argumentando lo contrario.

Dados los incansables intentos de la industria por socavar la implementación y la falta de recursos, voluntad política y capacidad de Estado,[13] es imperativo que los grupos de salud pública intensifiquen sus esfuerzos durante la redacción de los reglamentos de una ley para minimizar la presión de la industria y garantizar un alto cumplimiento y una implementación exitosa.[13] Esto se hizo realidad en México, ya que el apoyo de parte de los grupos de partes interesadas locales y extranjeros, parece haber influido en los tomadores de decisión para mantener varias disposiciones clave para el etiquetado frontal, incluida la adhesión al Modelo de Perfil de Nutrientes de la OPS, las porciones estandarizadas y la prohibición de los personajes animados al proporcionar evidencia científica y compartir lecciones aprendidas de otros países como Chile y Perú. Esfuerzos similares se han extendido a la implementación de etiquetado frontal en Uruguay y Argentina[4] y deben continuarse más esfuerzos para mejorar las alianzas con otros grupos de defensa a nivel transnacional para otros gobiernos que intentan aprobar e implementar el etiquetado frontal, como Brasil y Bolivia.

A pesar de este éxito, la industria de alimentos y bebidas aún pudo influir en los tomadores de decisión para debilitar varias disposiciones, incluida la autorización de declaraciones de propiedades saludables de grupos médicos respaldados por la industria, el debilitamiento del etiquetado de advertencia, los empaques colectivos, las advertencias de edulcorantes, entre otros. Estos cambios debilitados potencialmente

tuvieron efectos indirectos en términos de implementación, ya que la industria ha explotado lagunas en las regulaciones para continuar comercializando y publicitando sus personajes animados en otros medios y diseñando productos con doble cara para engañar a los consumidores en el punto de venta. Para evitar un patrón similar, los gobiernos deben prohibir que las empresas utilicen doble caras, implementar regulaciones complementarias para entornos de venta de alimentos al por menor, especialmente en puntos de venta en tiendas y supermercados, así como estrategias de marketing relacionadas, eliminando así las lagunas y obligando a los productores a cumplir con las regulaciones y no depender de los dueños de las tiendas. Dada la capacidad de la industria para continuar publicitando los personajes animados y los productos de sus marcas dirigidos a infancias en otros medios, como los espacios digitales, se deben fortalecer las regulaciones estrictas para reducir el poder y la exposición al marketing.

Las empresas de alimentos y bebidas también han intentado amenazar y desafiar legalmente el etiquetado frontal en México. Similar a la experiencia en el control del tabaco, estas representan tácticas de intimidación de la industria en un intento de bloquear, debilitar y retrasar las regulaciones de salud pública, especialmente en los países de ingresos bajos y medianos. [10, 12] Otros gobiernos pueden aprender de la experiencia de México que estas amenazas no necesariamente resultan en la anulación de las regulaciones implementadas.

Limitaciones

Dado que se presentaron 792 comentarios públicos, este estudio no realiza un análisis completo de los comentarios públicos, lo que limita la profundidad de los problemas planteados. Sin embargo, una de las fortalezas del estudio es capturar la amplitud de la información relacionada con la implementación del etiquetado frontal, incluida la aplicación, el monitoreo, las barreras y cómo la Norma se mantuvo igual y cambió según un resumen de los comentarios públicos. Además, este estudio es un relato descriptivo del proceso que no examinó el discurso o los diferentes intereses dentro del gobierno.

Conclusión

El éxito inicial en la implementación del etiquetado frontal en México fue el resultado de un proceso regulatorio inclusivo y participativo dedicado a mantener los avances en salud pública, el apoyo a la defensa de la salud local e internacional y el monitoreo continuo. Otros países que propongan y promulguen EFN podrían aprender de la experiencia mexicana para mantener las mejores prácticas científicamente probadas, contrarrestar las barreras de la industria y minimizar los retrasos en la implementación.

REFERENCIAS

1. World Cancer Research Fund. Diet, Nutrition, Physical Activity and Cancer: A Global Perspective: The Third Expert Report, May 2018. Available at: https://www.wcrf.org/dietandcancer. Accessed 20 April 2020.
2. World Health Organization. Implementing Nutrition Labelling Policies: A Review of Contextual Factors, 14 September 2021. Available at: https://www.who.int/publications/i/item/9789240035089. Accessed 20 November 2021.
3. Crosbie E, Gomes FS, Olvera J, *et al.* A Policy Study on Front-of-Pack Nutrition Labeling in the Americas: Emerging Developments and

Outcomes. *Lancet Reg Health Am* 2023;**18**:100400.
4. Ares G, Antunez L, Curutchet MR, *et al.* Immediate Effects of the Implementation of Nutritional Warnings in Uruguay: Awareness, Self-Reported Use and Increased Understanding. *Public Health Nutr* 2021;**24**:364-375.
5. Crosbie E, Carriedo A, Schmidt L. Hollow Threats: Transnational Food and Beverage Companies' Use of International Agreements to Fight Front-of-Pack Nutrition Labeling in Mexico and Beyond. *Int J Health Policy Manag* 2020.
6. Thow AM, Jones A, Hawkes C, *et al.* Nutrition Labelling Is a Trade Policy Issue: Lessons from an Analysis of Specific Trade Concerns at the World Trade Organization. *Health Promot Int* 2017.
7. Barquera S, Rivera JA. Obesity in Mexico: Rapid Epidemiological Transition and Food Industry Interference in Health Policies. *Lancet Diabetes Endocrinol* 2020;**8**:746-747.
8. Pedroza-Tobias A, Crosbie E, Mialon M, *et al.* Food and Beverage Industry Interference in Science and Policy: Efforts to Block Soda Tax Implementation in Mexico and Prevent International Diffusion. *BMJ Glob Health* 2021;**6**.
9. Bhatta DN, Bialous S, Crosbie E, *et al.* Exceeding Fctc Obligations: Nepal Overcoming Tobacco Industry Interference to Enact a Comprehensive Tobacco Control Policy. *Nicotine Tob Res* 2019.
10. Crosbie E, Thomson G, Freeman B, *et al.* Advancing Progressive Health Policy to Reduce Ncds Amidst International Commercial Opposition: Tobacco Standardised Packaging in Australia. *Glob Public Health* 2018;**13**:1753-1766.
11. White M, Barquera S. Mexico Adopts Food Warning Labels, Why Now? *Health Syst Reform* 2020;**6**:e1752063.
12. Crosbie E, Sosa P, Glantz SA. Defending Strong Tobacco Packaging and Labelling Regulations in Uruguay: Transnational Tobacco Control Network Versus Philip Morris International. *Tob Control* 2018;**27**:185-194.
13. Crosbie E, Sosa P, Glantz SA. The Importance of Continued Engagement During the Implementation Phase of Tobacco Control Policies in a Middle-Income Country: The Case of Costa Rica. *Tob Control* 2017;**26**:60-68.
14. Secretaría de Economía. Proyecto De Modificación a La Norma Oficial Mexicana Nom-051-Scfi/Ssa1-2010, Especificaciones Generales De Etiquetado Para Alimentos Y Bebidas No Alcohólicas Preenvasados- Información Comercial Y Sanitaria, Publicada El 5 De Abril De 2010., 11 October 2019. Available at: https://dof.gob.mx/nota_detalle.php?codigo=5575205&fecha=11/10/2019#gsc.tab=0. Accessed 27 October 2021.
15. Secretaría de Economía. Modificación a La Norma Oficial Mexicana Nom-051-Scfi/Ssa1-2010, Especificaciones Generales De Etiquetado Para Alimentos Y Bebidas No AlcohóLicas Preenvasados- InformacióN Comercial Y Sanitaria, Publicada El 5 De Abril De 2010, 27 March 2020. Available at: https://www.dof.gob.mx/2020/SEECO/NOM_051.pdf. Accessed 27 October 2021.

16. Knill C, Tosun J. Policy Making, January 2008. Available at: https://kops.uni-konstanz.de/bitstream/handle/123456789/3885/WorkingPaper2008_01.pdf. Accessed 10 December 2018.
17. Confederación de Cámaras Industriales de los Estados Unidos Mexicanos. Public Comment to the Official Mexican Standards (Nom-051-Scfi/Ssa1-2010), 6 December 2019. Available at: https://cofemersimir.gob.mx/expediente/23561/recibido/63296/B000195665. Accessed 7 February 2022.
18. Consejo Mexicano de la Industria de Productos de Consumo. Public Comment to the Official Mexican Standards (Nom-051-Scfi/Ssa1-2010), 12 November 2019. Available at: https://cofemersimir.gob.mx/expediente/23561/recibido/62999/B000194786. Accessed 7 February 2022.
19. Cámara de Diputados del H. Congreso de la Unión. Ley Federal De Protección Al Consumidor: Nueva Ley Publicada En El Diario Oficial De La Federación El 24 De Diciembre De 1992, 12 April 2019. Available at: https://www.diputados.gob.mx/LeyesBiblio/pdf_mov/Ley_Federal_de_Proteccion_al_Consumidor.pdf. Accessed 7 February 2022.
20. Grupo Jumex. Public Comment to the Official Mexican Standards (Nom-051-Scfi/Ssa1-2010), 10 December 2019. Available at: https://cofemersimir.gob.mx/expediente/23561/recibido/63394/B000195897. Accessed 7 February 2022.
21. El Poder del Consumidor. Public Comment to the Official Mexican Standards (Nom-051-Scfi/Ssa1-2010), 9 December 2019. Available at: https://www.cofemersimir.gob.mx/expediente/23561/recibido/63313/B000195712. Accessed 17 February 2022.
22. Instituto Nacional de Salud Pública. Public Comment to the Official Mexican Standards (Nom-051-Scfi/Ssa1-2010), 11 December 2019. Available at: https://cofemersimir.gob.mx/expediente/23561/recibido/63386/B000195867. Accessed 10 February 2022.
23. Coalición ContraPESO. Public Comment to the Official Mexican Standards (Nom-051-Scfi/Ssa1-2010), 9 December 2019. Available at: https://cofemersimir.gob.mx/expediente/23561/recibido/63328/B000195737. Accessed 17 February 2022.
24. Secretaría de Economía. Respuesta a Los Comentarios Recibidos Al Proyecto De ModificacióN a La Norma Oficial Mexicana Proy-Nom-051-Scfi/Ssa1-2010, Especificaciones Generales De Etiquetado Para Alimentos Y Bebidas No AlcohóLicas Preenvasados-InformacióN Comercial Y Sanitaria, Publicado El 11 De Octubre De 2019, 10 March 2020. Available at: https://www.dof.gob.mx/2020/SECO/Respuestas_PROY_NOM_051_SCFI_SSA1_2010.pdf. Accessed 3 February 2022.
25. Consejo Mexicano de la Carne. Public Comment to the Official Mexican Standards (Nom-051-Scfi/Ssa1-2010), 9 December 2019. Available at: https://cofemersimir.gob.mx/expediente/23561/recibido/63308/B000195701. Accessed 26 February 2022.
26. Ferrero de México. Public Comment to the Official Mexican Standards

(Nom-051-Scfi/Ssa1-2010), 25 November 2019. Available at: https://www.cofemersimir.gob.mx/expediente/23561/recibido/63109/B000195023. Accessed 7 February 2022.
27. Thow AM, Jones A, Schneider CH, *et al.* Global Governance of Front-of-Pack Nutrition Labelling: A Qualitative Analysis. *Nutrients* 2019;**11**.
28. Staff. Alimentación Saludable: ¿Empresas Reducen Tamaño De Empaques Para Eliminar Octógonos? Gestión. 24 March 2019: Available at: https://gestion.pe/economia/empresas/alimentacion-saludable-empresas-reducen-tamano-empaques-eliminar-octogonos-262264-noticia/. Accessed 3 March 2022.
29. The Hunger Project. Public Comment to the Official Mexican Standards (Nom-051-Scfi/Ssa1-2010), 9 December 2019. Available at: https://cofemersimir.gob.mx/expediente/23561/recibido/63314/B000195708. Accessed 5 June 2022.
30. World Public Health Nutrition Association. Public Comment to the Official Mexican Standards (Nom-051-Scfi/Ssa1-2010), 4 December 2019. Available at: https://cofemersimir.gob.mx/expediente/23561/recibido/63206/B000195368. Accessed 14 March 2022.
31. Global Health Advocacy Incubator. Public Comment to the Official Mexican Standards (Nom-051-Scfi/Ssa1-2010), 10 December 2019. Available at: https://www.cofemersimir.gob.mx/expediente/23561/recibido/63381/B000195861. Accessed 30 March 2022.
32. World Health Organization. Guiding Principles and Framework Manual for Front-of-Pack Labelling for Promoting Healthy Diet. Geneva, Switzerland, May 2019. Available at: https://www.who.int/nutrition/publications/policies/guidingprinciples-labelling-promoting-healthydiet.pdf?ua=1. Accessed 10 January 2020.
33. Stern D, Tolentino L, Barquera S. RevisióN Del Etiquetado Frontal: AnáLisis De Las Guías Diarias De AlimentacióN (Gda) Y Su ComprensióN Por Estudiantes De NutricióN En MéXico, May 2011. Available at: https://elpoderdelconsumidor.org/wp-content/uploads/2015/07/Etiquetado-Evaluación-GDA-por-Barquera-y-col.pdf. Accessed 18 February 2022.
34. United States Department of Agriculture Foreign Agricultural Service. Implementation of the New Mexican Regulation for Front-of Package Nutrition Label, 3 April 2020. Available at: https://apps.fas.usda.gov/newgainapi/api/Report/DownloadReportByFileName?fileName=Implementation%20of%20the%20New%20Mexican%20Regulation%20for%20Front-of-Package%20Nutrition%20Label%20_Mexico%20CIty%20ATO_Mexico_04-03-2020. Accessed 10 November 2021.
35. Secretaria de Gobernacion de Mexico. Acuerdo Por El Cual Se Establecen Los Criterios Para La Implementacion, Verificacion Y Vigilancia, Asi Como Para La Evaluacion De La Conformidad De La Modificacion a La Norma Oficial Mexicana Nom-051-Scfi/Ssa1-2010, 10 July 2020. Available at: https://www.dof.gob.mx/nota_detalle.php?codigo=5596558&fecha=10/07/2020. Accessed 20 March 2022.

36. De la Rosa E, Ochoa C. Multas Superiores a $700 Mil Por No Usar El Nuevo Etiquetado. Milenioz. 14 September 2020: Available at: https://www.milenio.com/negocios/multas-superiores-a-700-mil-por-no-usar-el-nuevo-etiquetado. Accessed 10 February 2022.
37. De la Rosa E. Asegura Profeco Que 99% De La Industria Ha Cumplido Con Nuevo Etiquetado En Alimentos. Milenio. 3 February 2021: Available at: https://www.milenio.com/negocios/profeco-asegura-99-industria-cumple-etiquetado. Accessed 5 December 2021.
38. El Poder del Consumidor. Todo Lo Que Debes Saber Sobre El Nuevo Etiquetado De Advertencia, 12 May 2021. Available at: https://elpoderdelconsumidor.org/2020/05/todo-lo-que-debes-saber-sobre-el-nuevo-etiquetado-de-advertencia/. Accessed 10 November 2021.
39. Comisión Federal para la Protección contra Riesgos Sanitarios. Manual De ModificacióN a La Norma Oficial Mexicana Nom-051-Scfi/Ssa1-2010: Especificaciones Generales De Etiquetado Para Alimentos Y Bebidas No AlcohóLicas Preenvasados., November 2020. Available at: https://www.gob.mx/cms/uploads/attachment/file/653810/Manual_NOM-051_versio_n_final.pdf. Accessed 4 March 2022.
40. Secretaria de Gobernacion de Mexico. Sesion Tecnica Nom-51: Comidas Preparadas, 26 July 2021. Available at: https://www.youtube.com/watch?v=Tiuz4ETOpv0. Accessed 15 November 2021.
41. Rueda R. Sancionan Sólo a 50 Empresas Por Incumplir Con Etiquetado. El Sol de Mexico. 22 January 2022: Available at: https://www.elsoldemexico.com.mx/mexico/sociedad/sancionan-solo-a-50-empresas-por-incumplir-con-etiquetado-7771264.html. Accessed 15 March 2022.
42. Staff. ¿Por Qué Decomisaron 380.000 Productos De Kellogg's En México? Los Angeles Times. 15 January 2022: Available at: https://www.latimes.com/espanol/mexico/articulo/2022-01-15/por-que-decomisan-380-000-productos-de-kelloggs-en-mexico. Accessed 30 January 2022.
43. Echeverría M. Conméxico Prevé Una 'Ola De Amparos' Por El Nuevo Etiquetado Frontal. Expansion. 1 October 2020: Available at: https://expansion.mx/empresas/2020/10/01/conmexico-preve-una-ola-de-amparos-por-el-nuevo-etiquetado-en-frontal. Accessed 20 June 2022.
44. Echeverría M. Amparos Y El Adiós Del Tigre Toño: El Saldo Del Etiquetado Frontal. Expansion. 1 April 2021: Available at: https://expansion.mx/empresas/2021/04/01/amparos-y-el-adios-del-tigre-tono-el-saldo-del-etiquetado-frontal. Accessed 22 June 2022.
45. United States Department of Agriculture Foreign Agricultural Service. Interinstitutional Agreement on Standard Nom-051 Fopl Compliance, 16 March 2021. Available at: https://apps.fas.usda.gov/newgainapi/api/Report/DownloadReportByFileName?fileName=Interinstitutional%20Agreement%20on%20Standard%20NOM-051%20FOPL%20Compliance_Mex

ico%20CIty%20ATO_Mexico_03-12-2021. Accessed 24 April 2022.

46. Rocha E. Bimbo Burla Nom-51 E Internautas Lo Hacen Viral. InformaBTL. 12 February 2021: Available at: https://www.informabtl.com/bimbo-burla-nom-51-e-internautas-lo-hacen-viral/. Accessed 10 November 2021.

47. Staff. Kellogg's: Creatividad Por Encima De La Ley. Excelsior. 13 May 2021: Available at: https://www.excelsior.com.mx/nacional/kelloggs-creatividad-por-encima-de-la-ley/1448658. Accessed 10 October 2021.

48. Rodriguez A. Bimbo, Lala Y Otras Empresas Combaten Nuevo Etiquetado Con Información En El Código De Barras. El Financiero. 10 February 2021: Available at: https://www.elfinanciero.com.mx/empresas/bimbo-lala-y-otras-empresas-combaten-nuevo-etiquetado-con-informacion-en-el-codigo-de-barras/. Accessed 5 December 2021.

49. Staff. El 50% De Los Mexicanos Entienden Mejor El Nuevo Etiquetado De Alimentos: Encuesta. Forbes Mexico. 16 October 2020: Available at: https://www.forbes.com.mx/noticias-encuesta-revela-que-50-de-los-mexicanos-entienden-mejor-el-nuevo-etiquetado-de-alimentos/. Accessed 3 November 2021.

50. Taillie LS, Bercholz M, Popkin B, *et al.* Changes in Food Purchases after the Chilean Policies on Food Labelling, Marketing, and Sales in Schools: A before and after Study. *Lancet Planet Health* 2021;**5**:e526-e533.

Tabla 1: Desarrollo y resultado de las Normas Oficiales Mexicanas para el etiquetado frontal nutricional del empaque en México (octubre 2019-marzo 2020)

Disposición	Proyecto de la NOM (octubre 2019)[14]	Argumentos en oposición (oct-dic 2019)	Argumentos de apoyo (oct-dic 2019)	NOM Final (Marzo 2020)[15]	Resultado
Prohibición de declaraciones de propiedades saludables	Prohibía todas las declaraciones de propiedades saludables y el uso de avales de asociaciones profesionales de la salud en productos con etiquetado de advertencias.	- Viola la Ley Federal del Consumidor Artículo 32 (establece los requisitos mínimos para el uso de avales) [17, 18]<br>- Descalifica la credibilidad de una entidad gubernamental (p.ej.,, Secretaría de Salud)[20]	-Toda la información bajo la Ley Federal de Protección al Consumidor debe estar libre de elementos confusos o engañosos y el Estado tiene interés legítimo en salud pública[21-23]<br>-Algunos avales de asociaciones médicas tienen conflictos de intereses (p. ej., la Federación Mexicana de Diabetes recibe fondos de Nestlé)[22]	Permite cualquier declaración o uso de avales de asociaciones profesionales de la salud solo en productos sin el etiquetado frontal de advertencia	-Sin cambios<br>-Explicación de la SE: Permitido de conformidad con la Ley Federal de Protección al Consumidor para promover el consumo informado<br>-(Victoria de la salud pública)
Etiquetado de advertencia para edulcorantes añadidos	Requería sellos de advertencia (octágonos negros) en productos que contengan edulcorantes agregados. El sello incluiría el texto "Contiene edulcorantes, evitar en niños"	-FDA y CODEX consideran que los edulcorantes son seguros[20, 25, 26]<br>- Plantea obstáculos innecesarios para el comercio y viola el Acuerdo de OTC [24]<br>- La redacción del etiquetado de advertencia restringe el consumo; excede la autoridad de la entidad reguladora[24]<br>-Dificulta la reformulación[26]	-Existe evidencia que indica el impacto dañino de los edulcorantes[21, 22]<br>-La visibilidad del etiquetado de advertencia es crucial, especialmente porque estos productos a menudo son publicitados para niños.	Requiere leyendas de advertencia (cuadros de texto más pequeños) en productos que contengan edulcorantes agregados. La redacción de la etiqueta se cambió a "Contiene edulcorantes. No recomendable en niños"	-Sin cambios<br>-Explicación de la SE: Permitido de conformidad con la Ley General de Salud Articulo 212 y basado en la evidencia que los edulcorantes pueden inculcar una preferencia por los sabores dulces entre los niños<br>-(Victoria de la industria)
Área de exhibición del etiquetado de advertencia (Etiquetado de advertencia simplificado)	Permitía que productos con un área de exhibición ≤20cm2 incluyeran solo un sello de advertencia simplificado (un octágono negro con el digito correspondiente a la cantidad de nutrientes críticos que contienen)	-El etiquetado frontal interfiere con la visibilidad de otros elementos en la etiqueta del producto[24]<br>- Productos con un área de exhibición <20cm están exentos de incluir información nutricional y, por lo tanto, deberían estar exentos del etiquetado frontal[24]	-Es indispensable establecer parámetros de área de exhibición más estrechos para los sellos de advertencia simplificados (es decir, disminuir el umbral a ≤10 cm2). La industria en Perú redujo el tamaño de sus productos para evadir los requisitos del etiquetado frontal[22]	Duplicó los parámetros y permitió que los productos con un área de exhibición ≤40 cm2 incluyeran solo un sello de advertencia simplificado (un octágono negro con el dígito correspondiente a la cantidad de nutrientes críticos que contienen).	-Cambió<br>- Explicación de la SE: Las dimensiones propuestas por el Proyecto de NOM obstruirían la visibilidad del etiquetado frontal<br>-(Victoria de la industria)
Etiquetado de advertencia en empaques colectivos	Requería que todos los productos individuales comercializados en empaques colectivos exhibieran el etiquetado frontal correspondiente (además de aquellos en el empaque colectivo)	-Productos en empaques colectivos requieren incluir la leyenda "No etiquetado para su venta individual," y por tanto deben ser exentos de los requisitos del etiquetado frontal[25]	N/A	Requiere que solo el empaque colectivo incluya el etiquetado frontal	- Cambió<br>- Explicación de la SE: Ninguna<br>-(Victoria de la industria)

Declaración libre de EFN "este producto no contiene sello ni leyendas"	Prohibía que los productos indicaran que son libres del etiquetado frontal	-Viola la Ley Federal de Protección del Consumidor Articulo (requiere información clara y precisa sobre el contenido, la composición y las características del producto)[25]	-Los productos pueden usar estas declaraciones para hacer parecer sus productos más saludables de lo que son realmente. La industria usa esta estrategia en Perú [24]	Permite que los productos sin etiquetado frontal incluyan el texto "No contiene sellos ni leyendas" en su empaque principal	-Cambió<br>- Explicación de la SE: Ninguna<br>-(Victoria de la industria)
Posicionamiento del etiquetado de advertencia en productos con área de exhibición principal ≤60cm	Requería que todos los productos, a pesar de su tamaño incluyeran los sellos de advertencia (octágonos) correspondientes estrictamente en l esquina superior derecha de su área de exhibición principal	-Falta evidencia para justificar el uso de etiquetado frontal de advertencia[24]<br>-El posicionamiento del etiquetado de advertencia es irrelevante[24, 25]<br>- Los productores deben poder colocar el etiquetado de advertencia frontal en cualquier parte del área de exhibición principal del producto[18]	-Con base en la experiencia de Chile, la regulación debería establecer una zona protegida alrededor del etiquetado de advertencia para evitar que los productores incluyan mensajes adicionales[24]	Permite que productos con un área de exhibición principal ≤60cm2 incluyan los elementos del etiquetado frontal correspondientes en cualquier parte del área de exhibición principal	- Cambió<br>- Explicación de la SE: El posicionamiento del etiquetado de advertencia en el área de exhibición principal tiene como objetivo promover la toma de decisiones informadas.<br>-(Victoria de la industria)
Modelo de Perfil de Nutrientes	Utilizó el Modelo de Perfil de Nutrientes de la Organización Panamericana de la Salud (OPS) como criterio rector para la implementación del etiquetado frontal	-Se deberían incrementar los umbrales para los nutrientes criticos[24]<br>-Se deberían modificar las porciones estandarizadas propuestas[24]<br>-Se debería modificar el lenguaje utilizado en los sellos de advertencia de "Exceso" a "Alto en"<br>-Se debería de modificar el Modelo de Perfil de Nutrientes ya que viola la Ley General de Salud[25]<br>Dificulta la reformulación[20, 25]	-Este modelo está basado en la evidencia y diseñado específicamente para abordar las necesidades de salud críticas de la región[21, 22, 30, 31]	Utiliza el Modelo de Perfil de Nutrientes de la OPS como criterio rector para la implementación del etiquetado frontal	-Sin cambios<br>-Explicación de la SE: La OPS participó en los grupos de trabajo que revisaron las presentaciones de comentarios públicos y presentó pruebas suficientes para fundamentar el uso de este modelo<br>-(Victoria de la salud pública)
Porciones estandarizadas	Establecía una porcion estandarizada de 100g o 100 ml como referencia para toda la información nutricional en productos preenvasados	-Los nutrientes críticos deberían declarase por porción "tipica"[18, 25]	- Promueve la comprensión del consumidor y permite una comparación más fácil del valor nutricional entre productos[21-23, 29, 31]	Estableció una porcion estandarizada de 100g o 100 ml como referencia para toda la información nutricional en productos preenvasados	-Sin cambios<br>-Explicación de la SE: Permitido de conformidad con la Ley General de Salud<br>-(Victoria de la salud pública)
Prohibición de personajes animados	Prohibía todos los personajes animados y la publicidad dirigida a niños en productos con etiquetado de advertencia	-Viola leyes y acuerdos locales e internacionales (p.ej., la Ley Federal del Derecho de Autor, Tratado de la Organización Mundial de la Propiedad Intelectual sobre Derecho de Autor)[17, 18, 25, 26]	-Medida basada en evidencia, respaldada por entidades como UNICEF y la OMS [21, 23, 29-3]<br>-No infringe ninguna ley de comercio internacional[24]	Prohíbe todos los personajes animados y la publicidad dirigida a niños en productos con etiquetado de advertencia	-Sin cambios<br>-Explicación de la SE: El uso de personajes y otros elementos publicitarios promueven el consumo de productos que presentan un riesgo para la salud de los niños

					-(Victoria de la salud pública)
EFN: Etiquetado frontal nutricional<br>SS: Secretaría de Economía<br>NOM: Normal Oficial Mexicana<br>Grupos de apoyo a la norma: Instituto Nacional de Salud Pública (INSP), Contra Peso, El Poder del Consumidor, Hunger Project, Global Health Advocacy Incubator y World Public Health Nutrition Association.<br>Grupos en oposicion a la norma: Consejo Coordinador Empresarial (CCE), Mead Johnson Nutrition, Consejo Mexicano de la Carne, Confederación de Cámaras Industriales (CONCAMIN), Consejo Mexicano de la Industria de Productos (CONMEXICO), los cuales representan a algunas de las empresas de alimentos y bebidas más grandes del mundo, incluido Nestlé, Kellogg, Hershey, Danone, Unilever, Mars, Coca Cola, PepsiCo, y Grupo Bimbo.					
